# Multimodal Neuroimaging in Schizophrenia: Description and Dissemination

**DOI:** 10.1007/s12021-017-9338-9

**Published:** 2017-08-15

**Authors:** C. J. Aine, H. J. Bockholt, J. R. Bustillo, J. M. Cañive, A. Caprihan, C. Gasparovic, F. M. Hanlon, J. M. Houck, R. E. Jung, J. Lauriello, J. Liu, A. R. Mayer, N. I. Perrone-Bizzozero, S. Posse, J. M. Stephen, J. A. Turner, V. P. Clark, Vince D. Calhoun

**Affiliations:** 1 The Mind Research Network, 1101 Yale Blvd NE, Albuquerque, NM 87106 USA; 2 Department of Radiology, University of New Mexico SOM, Albuquerque, NM USA; 3 Advanced Biomedical Informatics, LLC, Coralville, IA USA; 4 Department of Psychiatry, University of Iowa, SOM, Iowa City, IA USA; 5 Department of Psychiatry, University of New Mexico SOM, Albuquerque, NM USA; 6 New Mexico VA Health Care System, Albuquerque, NM USA; 7 Department of Neurosurgery, University of New Mexico SOM, Albuquerque, NM USA; 8 Department of Psychiatry, University of Missouri Health Care, Columbia, MO USA; 9 Department of Neurosciences, University of New Mexico SOM, Albuquerque, NM USA; 10 Department of Neurology, University of New Mexico SOM, Albuquerque, NM USA; 11 Department of Electrical and Computer Engineering, Albuquerque, NM USA; 12 Department of Physics and Astronomy, Albuquerque, NM USA; 13 Department of Psychology, Georgia State University, Atlanta, GA USA; 14 Department of Psychology, University of New Mexico, Albuquerque, NM USA

**Keywords:** Neuroimaging, Schizophrenia, fMRI, MEG, Magnetoencephalography, Spectroscopy, Genetics, Sensory gating, Multimodal integration, Memory, Transverse patterning, DTI, ICA, COINS, COBRE, MATRICS

## Abstract

In this paper we describe an open-access collection of multimodal neuroimaging data in schizophrenia for release to the community. Data were acquired from approximately 100 patients with schizophrenia and 100 age-matched controls during rest as well as several task activation paradigms targeting a hierarchy of cognitive constructs. Neuroimaging data include structural MRI, functional MRI, diffusion MRI, MR spectroscopic imaging, and magnetoencephalography. For three of the hypothesis-driven projects, task activation paradigms were acquired on subsets of ~200 volunteers which examined a range of sensory and cognitive processes (e.g., auditory sensory gating, auditory/visual multisensory integration, visual transverse patterning). Neuropsychological data were also acquired and genetic material via saliva samples were collected from most of the participants and have been typed for both genome-wide polymorphism data as well as genome-wide methylation data. Some results are also presented from the individual studies as well as from our data-driven multimodal analyses (e.g., multimodal examinations of network structure and network dynamics and multitask fMRI data analysis across projects). All data will be released through the Mind Research Network’s collaborative informatics and neuroimaging suite (COINS).

**Electronic supplementary material:**

The online version of this article (doi:10.1007/s12021-017-9338-9) contains supplementary material, which is available to authorized users.

## Introduction

The purpose of this paper is to announce the release of a large multimodal neuroimaging dataset on chronic schizophrenia patients and healthy controls [e.g., functional MRI, diffusion tensor imaging (DTI), proton MR spectroscopic imaging (^1^H–MRS), and magnetoencephalography (MEG) data] along with clinical, genetic, and neurocognitive assessments. Data were acquired from an NIH-supported COBRE (Centers of Biomedical Research Excellence) Phase I grant (2P20GM103472). Here we describe the projects that generated this large multimodal dataset for dissemination. An overview of the hypotheses motivating these projects and types of multimodal data to be released are presented in the Introduction, followed by details of each experimental protocol and a synopsis of results in the Methods & Results Section, followed by a plan for releasing the data in the Information Sharing section.

The multimodal data to be released were acquired across four hypothesis-driven projects with a goal of imaging the same sample of approximately 200 volunteers within each project. This included 100 patients with schizophrenia (SPs) and 100 healthy controls (HCs). In addition, analysis using data-driven approaches developed as part of the COBRE (e.g., multitask fMRI data analysis across projects) are presented. Schizophrenia is a serious illness that is primarily characterized by delusions, hallucinations, and disorganized speech. However, most patients also have cognitive dysfunction, resulting in problems with social and interpersonal interactions (Green et al. 2004, Green and Nuechterlein 2004). This dysfunction led to its original conception as an early form of dementia (dementia praecox) by Kraepelin (1896). Although current drug regimens are mostly successful at controlling the psychotic symptoms of the disorder (~20–30% do not respond adequately), cognitive and social functions generally remain impaired. Our Mind Research Network (MRN) COBRE team views schizophrenia as a disorder characterized by abnormalities in structural, functional, and effective connectivity between cortical and subcortical brain regions, producing abnormalities in information processing across distributed brain circuits. The novel combination of non-invasive neuroimaging techniques used here provided an unprecedented view of the neuronal pathologies that underlie the core cognitive dysfunctions of schizophrenia.

**Fig. 1:**
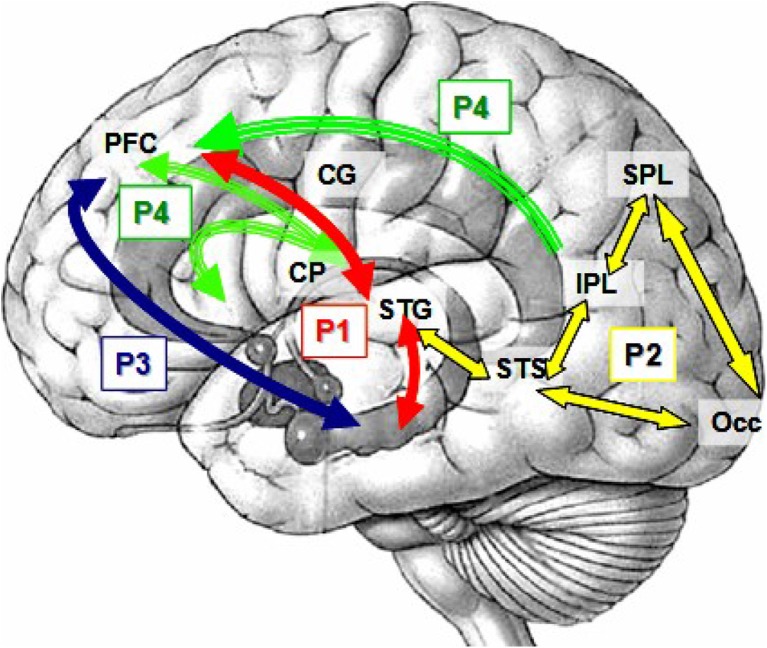
Complementary networks investigated in the four projects. Project 1 (P1, red arrows) explored the functional connectivity between the superior temporal gyrus (STG), hippocampus and prefrontal cortex (PFC) during auditory sensory gating. Project 2 (P2, yellow) examined the integration of simultaneously presented visual and auditory information in primary sensory and secondary cortical regions such as the occipital lobe (Occ), superior and inferior parietal lobe (SPL and IPL, respectively), the STG and the superior temporal sulcus (STS). The coherence between PFC and a hippocampal circuit was studied in Project 3 during a visual transverse patterning task (P3, blue). Project 4 (P4, green) examined structural connectivity between the cingulate gyrus (CG), PFC and caudate-putamen (CP)

Our primary scientific hypothesis was that schizophrenia affects distributed circuits that appear to be “disconnected” or poorly integrated. This, in turn, results in generalized cognitive dysfunction (Bullmore et al. 1997, Stephan et al. 2006). Similarly, in a 2004 NIH sponsored study, a panel of experts agreed that basic sensory processes, attention, memory, concept formation and problem solving abilities (i.e., intelligence) were among the top cognitive deficits that detrimentally affected patients with schizophrenia (Kern et al. 2004). Each of the four individual projects of this COBRE grant examined a portion of this hierarchy of sensory/cognitive processes, as identified by the expert NIH panel, as major domains of cognitive dysfunction. Thus, the four projects spanned a range of perceptual and cognitive processes (see Figure 1). This began with an examination of a basic inhibitory process during an auditory sensory gating task (Project 1—Dr. Mayer, PI—red arrows), multisensory integration during the simultaneous presentation of auditory-visual stimuli (Project 2—Dr. Stephen, PI—yellow arrows), memory integration during transverse patterning (Project 3—Dr. Hanlon, PI—blue arrows) and general cognitive impairment (Project 4—Dr. Jung, PI—green arrows). Note that the networks are complementary and build on each other while the anatomic localization of these functions has considerable overlap.

### Overview of the 4 Hypothesis-Driven Projects

Each of the projects addressed a specific domain of cognitive dysfunction and carefully probed the basis of impairments at different hierarchical levels. The resultant dataset is, to our knowledge, unique in that it includes multiple MRI and MEG measures on the same patients and controls, performing multiple tasks. All projects collected multimodal neuroimaging data on *up to* 100 of the same SPs and *up to* 100 HCs and a centralized data processing stream was developed and made available to the projects. Please note, however, that while there was significant overlap in participants tested in these projects, no project obtained data from all 200 participants, given the logistical challenges associated with acquiring approximately 7 h of multimodal imaging and neuropsychological assessments across visits. Table 1 shows the number of subjects that were shared among each of the projects.

**Table 1:** Numbers of participants for each project and task. Top portion—structural MR scans acquired on all SP and HC available to all projects. Bottom portion—Projects 1–3: functional scans (fMRI and MEG) acquired by some of the projects and for some of the SP and HC. Project 4: Structural and spectroscopy scans

I. Structural Scans		
Structural MRI (sMRI)	HC = 200 SP = 224 (2 scans each)	
II. Project Specific Scans	fMRI (N)	MEG (N)
Project 1: Auditory Gating/Orienting and Multisensory Cognitive Control		
Task 1—Gating In/Out	HC = 65 SP = 58	
Task 2—Orienting/Inhibition	HC = 66 SP = 57	HC = 25 SP = 30
Task 3—Resting State	HC = 94 SP = 115	HC = 22 SP = 24
Task 4—Multisensory Cognitive Control (Visual/auditory)	HC = 27 SP = 55	
Task 5—Multisensory Detection (Visual/auditory)	HC = 27 SP = 60	
Project 2: Multisensory Integration (auditory and visual)		
Auditory alone (near)	HC = 67 SP = 68	HC = 67 SP = 68
Visual alone (near)	HC = 67 SP = 68	HC = 67 SP = 68
Auditory/Visual (near-synch)	HC = 67 SP = 68	HC = 67 SP = 68
Auditory/Visual (near-asynch)	HC = 67 SP = 68	HC = 67 SP = 68
Auditory alone (far)	HC = 67 SP = 68	HC = 67 SP = 68
Visual alone (far)	HC = 67 SP = 68	HC = 67 SP = 68
Auditory/Visual (far-synch)	HC = 67 SP = 68	HC = 67 SP = 68
Auditory/visual (far-asynch)	HC = 67 SP = 68	HC = 67 SP = 68
Resting State		HC = 36 SP = 37
Project 3: Transverse Patterning (visual)		
Task 1—Elemental Control (verbal)	HC = 61 SP = 47	HC = 60 SP = 49
Task 2—TP (verbal)	HC = 61 SP = 47	HC = 60 SP = 49
Task 3—Elemental Control (nonverbal)	HC = 61 SP = 47	HC = 60 SP = 49
Task 4—TP (nonverbal)	HC = 61 SP = 47	HC = 60 SP = 49
Project 4: No Experimental Tasks		
Diffusion Tensor Imaging (DTI)	HC = 96 SP = 97	
Proton MR Spectroscopy (^1^H–MRS)	HC = 69 SP = 62	

There is general consensus in the schizophrenia literature that: 1) widespread, generalized deficits exist and appear to be related to disconnection between several regions; 2) deficits exist at both basic sensory and higher levels of functioning; 3) research can examine these disconnections but the problem is most likely more complex than one study can fully probe; and 4) multimodal imaging may provide complementary information (location, timing, content). However, there is no consensus on the primary sites of disconnection. Our four COBRE projects focused on anatomical and functional examples of cortico-cortical and cortico-subcortical disconnections in hemodynamic functioning (fMRI: Projects 1, 2 and 3), in the temporal dynamics of activity between these regions (MEG: Projects 1, 2 and 3), at the morphometric level (Project 4), and in white matter integrity (DTI: Projects 2, 3, and 4). In addition, Project 4 utilized MR Spectroscopic Imaging (^1^H–MRS) to examine metabolites of interest [e.g. N-acetylaspartate (NAA)] to potentially identify regions with primary neuronal damage.

#### Project 1: Multimodal Imaging of Auditory Pre-Attentive/Attentive and Cognitve Control Processes in SP (Andrew Mayer, PI)

This project acquired fMRI data from 4 task activation paradigms and MEG data from 1 task activation paradigm ranging from pre-attentive processes to multisensory cognitive control. Multiple lines of research have consistently documented auditory sensory processing deficits in SP. One such deficit which has been previously examined in SP is poor sensory gating; i.e., there is a failure to inhibit the electrophysiological response for the second of two rapidly presented stimuli in SP compared to HC (Adler et al. 1982, Freedman et al. 1987). This effect is typically reported in terms of a gating ratio comparing the amplitude of the response for the first (S1) and second (S2) stimulus (S2/S1*100). Poor sensory gating has been characterized as not only a deficit in selective attention and/or in the formation of memory traces, but also as a useful bio-marker of the cognitive and social dysfunction that is typically observed in SP (Cullum et al. 1993, Lijffijt et al. 2009). Electroencephalographic (EEG) and MEG studies of sensory gating have implicated the temporal lobes, including the superior temporal gyrus, as the most likely neuronal generator of the sensory gating deficit (Hanlon et al. 2005a, Huang et al. 2003, Thoma et al. 2003, 2005). Although other invasive neuroimaging techniques suggest a role for the hippocampus and prefrontal cortex in sensory gating (Boutros et al. 2005, Freedman et al. 1996, Grunwald et al. 2003, Korzyukov et al. 2007), neither EEG nor MEG studies have revealed hippocampal activation. While this negative result may be reflective of a true lack of hippocampal activation in gating, it may also be secondary to technical limitations with EEG and MEG, such as limits in spatial resolution, failure to examine appropriate time epochs, and/or difficulties localizing sources in deep structures under certain conditions. In contrast, fMRI is not restricted by these limitations, suggesting that it may be an alternative modality for studying the entire neuronal network underlying the gating deficit, including mesial temporal and prefrontal sources. In this protocol, SP and HC were presented with identical clicks, identical tones, and non-identical tones while undergoing functional imaging. It was predicted that a double dissociation of functioning would be observed with SP exhibiting higher gating ratios for the identical tones and clicks conditions and lower gating ratios for the non-identical tones condition, whereas HC would exhibit the opposite pattern. The functional data from these conditions were directly compared and correlated with behavioral and neuropsychological measures to further quantify the relationship between brain function and the observed clinical pathology that characterizes this basic inhibitory failure. Finally, since impaired sensory gating is thought to be an endophenotypical marker for schizophrenia, the genetic contribution to our measures of electrophysiological and hemodynamic functioning in both HC and SP were investigated as well. Data recorded from other tasks within this project included an auditory cueing paradigm that measured both exogenous orienting and inhibition of return and multisensory cognitive control and detection. A multisensory cognitive control paradigm was used to determine if deficits in SP result from dysfunction within the cognitive control network (CCN; top-down) and/or unisensory cortex (bottom-up), using congruent or incongruent multisensory (auditory and visual) numeric stimuli.

#### Project 2: Multisensory Integration in SP using MEG and fMRI (Julia Stephen, PI)

The goal of this project was to study the neural mechanisms underlying multisensory integration of auditory and visual stimuli in SP and HC using MEG and fMRI combined with structural MRI (sMRI). Integration of multisensory information is critical to understanding our environment and provides an important bridge between simple sensory perception and more complex cognitive tasks. That is, the integrity of a larger, distributed neural circuit can be evaluated without engaging complex cognitive functions, where issues of strategy, for example, can confound the results. It has been established that multisensory stimulation generally improves reaction times and performance (based on percent correct) relative to unisensory stimulation at levels that are difficult to perceive (Stein et al. 1993), and more recent results indicate that multisensory facilitation can be observed across the perception spectrum (Stanford and Stein 2007). Some reports have shown that SP have slower reaction times to multisensory stimuli than HC [e.g., (Williams et al. 2010)]. Since the association areas of the temporal lobe are necessary for integration of auditory and visual information (Schroeder and Foxe 2002, Schroeder et al. 2001), altered multisensory processing is consistent with the findings that SP show functional and anatomical differences in the temporal lobes [e.g., (Patterson et al. 2008)]. In this project, auditory and visual multisensory integration was studied using an ecologically valid paradigm that simulates the sight and sound of a soccer ball bouncing either near or far from the subject. The stimuli were presented as auditory alone, visual alone, and synchronous auditory/visual conditions, to which subjects responded with a button press. Based on known deficits in the temporal lobe, it was hypothesized that the responses to combined auditory/visual stimuli would be impaired in SP relative to HC. In addition, due to a reported deficit in the visual dorsal stream in SP (Butler et al. 2007, Butler et al. 2005, Doniger et al. 2002, Foxe et al. 2001, 2005, Kim et al. 2006, 2005, O'Donnell et al. 1996, Schechter et al. 2006, 2005), a neural circuit sensitive to the spatial location of objects, it was hypothesized that there would be a difference in visual responses to the near versus far stimuli in SP, relative to HC. These deficits may be related to unisensory deficits and/or to difficulties in synchronizing activity across widespread regions connected within the circuit which will likely be seen differently in MEG versus fMRI measures, given the complementary nature of these measurement techniques. The long term goal was to identify the local cortical deficits, as well as the deficits in cortical networks that lead to the abnormalities observed in SP. Knowledge about these deficits should permit the evaluation of relationships with neurochemical abnormalities, ultimately leading to more targeted pharmacological interventions.

#### Project 3: Fronto-temporal Coherence: A Test of the Disconnection Hypothesis in SP (Faith Hanlon, PI)

Prefrontal cortex (PFC) and hippocampal structures play a central role in working memory and relational memory impairments exhibited in SP (Goldman-Rakic 1994, Hanlon et al. 2005a, 2006, Honey and Fletcher 2006, Wolf et al. 2009). These PFC and hippocampal functional deficits have traditionally been attributed to properties of the cortical structures themselves. However, an alternative view is that the deficits are due to the disconnection between these structures in SP (Friston 1999, 2002, Friston and Frith 1995, Johnson 2006). Project 3 tested this disconnection hypothesis; that is, there is a functional disconnection between frontal and temporal cortices due to an abnormal anatomical connection of the underlying white matter tract, uncinate fasciculus, that links the two. The most striking evidence supporting this abnormal fronto-temporal connectivity in SP is found in neuroanatomical studies that describe abnormalities in the uncinate fasciculus (Burns et al. 2003, Kubicki et al. 2002, Park et al. 2004). Despite this finding of abnormal anatomical connectivity, the relationship between functional and anatomical connectivity of this PFC-hippocampal network has not been sufficiently assessed. The focus of this project was on examining the functional and anatomical connectivity in SP and HC using MEG and fMRI during a working-relational memory task, transverse patterning or TP (Burns et al. 2003, Hanlon et al. 2012, 2011, 2003, 2005b, Kubicki et al. 2002, Park et al. 2004). TP is very similar to the childhood-game “Rock, Paper, Scissors.” In TP, subjects chose between stimuli presented in pairs, with the correct choice being a function of the specific pairing. To complete the task the subject must discover, encode, and maintain the distinct relationships among the stimuli, thus requiring working memory and relational memory integration. Fronto-temporal functional connectivity was examined via assessing the temporal correlation, or coherence between the PFC (BA 9 and BA 10) and hippocampus during TP performance. In addition, anatomical connectivity using fractional anisotropy (FA) measures derived from DTI of the uncinate fasciculus was also evaluated. Finally, the functional/structural relationships between PFC and hippocampus, memory function, overall functioning, and clinical symptomatology were examined. Understanding these relationships could potentially lead to treatments or therapies aimed at improving memory, thereby improving overall functioning and clinical symptoms.

#### Project 4: Fronto-Subcortical Disconnection underlying Neurocognitive Dysfunction in SP (Rex Jung, PI)

This project used 3 T MRI to investigate whether general cognitive functioning in SP is related to circuit level white matter (WM), metabolic, and volumetric changes in subcortical gray matter (GM) and WM regions suggestive of fronto-subcortical disconnection. The available neuroscientific literature in HC, including functional (i.e., fMRI, positron emission tomography) and biochemical/structural (i.e., ^1^H–MRS, DTI, voxel-based morphometry or VBM) neuroimaging paradigms, show a striking consensus suggesting that variations in a distributed network predict individual differences found on intelligence and reasoning tasks in HC (Jung and Haier 2007). This parieto-frontal integration theory, or P-FIT model includes the dorsolateral PFC (BAs 6, 9, 10, 45, 46, 47), the inferior (BAs 39, 40) and superior (BA 7) parietal lobule, the anterior cingulate (BA 32), and regions within the temporal (BAs 21, 37) and occipital (BAs 18, 19) lobes. The main structural brain abnormality established in SP research is modest lateral ventricle enlargement indicative of atrophy (Chua and McKenna 1995), a finding established in the very first report utilizing computerized tomography (Johnstone et al. 1976), and suggestive of WM atrophic changes. Since then various brain abnormalities have been identified in SP, both cortically and subcortically, including gross morphological changes, reduced metabolism, and cellular pathology. This diffuse pathology has led some to hypothesize that SP is “a disease of neuronal connectivity,” arising from abnormalities at the level of the neuron and myelin (Andreasen 2000). Evidence for WM abnormality in SP includes structural oligodendrocyte abnormalities (Hof et al. 2002, 2003, Orlovskaya et al. 1997, 1999, Uranova et al. 2001), myelin protein alterations (Chambers and Perrone-Bizzozero 2004), myelin gene polymorphisms (Liu et al. 2005, Novak et al. 2002, Peirce et al. 2006, Qin et al. 2005, Tan et al. 2005, Wan et al. 2005), and altered expression of genes involved in the formation and maintenance of myelin sheaths as well as oligodendrocyte survival and proliferation revealed by DNA microarray methods (Bunney and Bunney 2000, Goldman-Rakic and Selemon 1997, Hakak et al. 2001). The simultaneous utilization of DTI, ^1^H–MRS, and sMRI allowed us to relate WM abnormalities to both cortical and subcortical metabolic and morphological changes that, in turn, may underlie ongoing neurocognitive decline in SP. Since SP was hypothesized to be a disorder that involves the integration of information among distributed brain circuits, Project 4 investigated: 1) whether fronto-subcortical network abnormalities are present in SP; 2) whether cognitive functioning in SP is related to circuit level dysfunction; and 3) whether regions beyond the identified brain circuits contribute significantly to broad cognitive functioning deficits characteristic of SP. A hierarchical approach was explored, in which cognitive functioning was predicted by cortical thinning, WM microstructure changes, and metabolic changes within discrete fiber tracts in SP. Also tested was the hypothesis that ongoing neurocognitive impairment in SP is related to chronic disconnection of fronto-subcortical circuits, reflected in morphological contraction of discrete frontal lobe regions critical to higher cognitive functioning. Finally, we explored whether specific genes associated with the formation, development, and functioning of myelin are associated with circuit level dysfunction in SP. No research to date has simultaneously explored diffusivity, metabolic, and morphometric abnormalities within these critical brain circuits as they relate to neurocognitive dysfunction in SP. We see this as a critical line of investigation that will help us understand and potentially intervene in the ongoing cognitive dysfunction that currently limits the ability of treated patients to return to more normal occupational and social functioning.

## Methods & Results

An overview of participant characteristics as well as data acquisition parameters common across the four projects using structural/functional imaging measures is presented here. Details of each project’s experimental protocols are discussed separately under each project description. Table 1 contains a breakdown of the number of SP and HC involved in each task and the types of data acquired for each task. All structural imaging, shared across projects, were acquired under Project 4. All studies were approved by the institutional review board (IRB) of the University of New Mexico (UNM) and all subjects provided written informed consent.

### Subject Recruitment and Evaluation

Patients were recruited primarily from the UNM Psychiatric Center and secondarily from the Raymond G. Murphy Veterans Affairs Medical Center, given the investigators’ clinical appointments. Some were recruited from other psychiatric clinics in the Albuquerque metropolitan area as well. Inclusion criteria for patient selection included diagnosis of schizophrenia or schizoaffective disorder between 18 and 65 years of age. Each SP completed the Structured Clinical Interview for DSM-IV Axis I Disorders [SCID--(First et al. 1995a, b)] for diagnostic confirmation (consensus was reached by two research psychiatrists using the SCID-DSM-IV-TR, patient version) and evaluation for co-morbidities. SP had to demonstrate retrospective and prospective clinical stability to be included in this investigation.

Because multiple neuroimaging sessions were required from each subject on different days, a concerted effort to prevent meaningful variation in the SP’s critical state variables (e.g., symptoms, medication dose, neurological side-effects) was built into the design of the study. Hence SP were seen weekly by the study clinicians during the period of data collection. The study clinicians and staff were responsible for subject recruitment, determining that subjects met clinical stability criteria, genetic testing, and administering all clinical assessments and standardized cognitive batteries to subjects. Standard symptom ratings [e.g., Positive and Negative Syndrome Scale (PANSS) (Kay et al. 1987)] as well as neurological side-effects [e.g., akathisia—(Barnes 1989); parkinsonism—(Simpson and Angus 1970); and tardive dyskinesia—(Schooler and Kane 1982)], were collected within one week of every neuroimaging assessment. The COBRE Stability Clinic determined retrospective stability from relevant psychiatric records documenting that no change in symptomatology or type/dose of psychotropic medications occurred during the three months prior to the referral. This Clinic assessed prospective stability during three consecutive weekly visits and during each imaging assessment. Prospective stability was defined as no change in clinical symptoms > two points from the positive symptom items on the PANSS, no score of “worse” or “much worse” on the Clinical Global Impression Scale (CGI) (Guy 1976), no suicidal or violent ideation, and no psychiatric or medical hospitalizations. The doses of antipsychotic medications were converted to olanzapine equivalents to estimate medication load (Gardner et al. 2010). Subjects with a history of neurological disorders including head trauma (loss of consciousness greater than 5 min), mental retardation or history of active substance dependence or abuse (except for nicotine) within the past year were excluded (i.e., history of dependence on phencyclidine (PCP), amphetamines or cocaine, or history of PCP, amphetamine, or cocaine use within the last 12 months). All subjects had a negative toxicology screen for drugs of abuse at the start of the study.

HCs were recruited from the same geographic location via IRB-approved advertisement and completed the SCID-Non-Patient Edition to rule out Axis I conditions (First et al. 1995a, b). Additional exclusion criteria for HCs included a current or past psychiatric disorder (with the exception of one lifetime major depressive episode), head trauma with a loss of consciousness greater than 5 min, recent history of substance abuse or dependence, depression or antidepressant use within the past 6 months, lifetime antidepressant use of more than one year, and history of a psychotic disorder in a first-degree relative.

SP and HC groups were similar in age (37.9 ± 14 vs. 37.5 ± 11.8) and male/female proportion (81/19 vs. 72/26), respectively. The SPs had been chronically ill (age of first psychotic symptoms 21.2 ± 8) and had lower education compared to HCs (3.8 ± 1.4 vs. 4.6 ± 1.3, respectively; 3 = high school graduate or equivalent, 4 = some college). All participants refrained from smoking for at least one hour before scanning.

### MRI/fMRI data collection

EPI slices were collected in sequential ascending order on a Siemens 3 T TIM Trio scanner, located at MRN, using a 12-channel head coil. A sagittal gradient echo scout image through the midline was obtained to prescribe oblique axial image slices parallel to the anterior-posterior commissure (AC-PC) line. Oblique slices were used to minimize the orbitofrontal susceptibility artifact (Deichmann et al. 2003). High resolution T1-weighted images were acquired with a 5-echo multi-echo MPRAGE sequence [TE (echo times) = 1.64, 3.5, 5.36, 7.22, 9.08 ms, TR (repetition time) = 2.53 s, TI (inversion time) = 1.2 s, 7^○^ flip angle, number of excitations (NEX) = 1, slice thickness = 1 mm, FOV (field of view) = 256 mm, resolution = 256 × 256] for region of interest analyses and spatial normalization. All fMRI data were collected using a conventional single-shot, gradient-echo echoplanar pulse sequence with lipid suppression [TR = 2000 ms; TE = 29 ms; flip angle = 75^○^; FOV = 240 mm; matrix size = 64 × 64; 33 slices; voxel size: 3.75 × 3.75 × 4.55 mm]. The first image of each run was eliminated to account for T1 equilibrium effects.

### Diffusion MRI (dMRI) Data Collection

dMRI data were collected along the AC-PC line, throughout the whole brain, with FOV = 256 × 256 mm, 128 × 128 matrix, 72 slices with a slice thickness of 2 mm (isotropic 2 mm resolution), NEX = 1, TE = 84 ms and TR = 9000 ms. A multiple-channel radiofrequency (RF) coil was used, with GRAPPA (X2), 30 gradient directions with b = 800 s/mm^2^. The b = 0 experiment was repeated five times (Jones et al. 1999), and equally interspersed between the 30 gradient directions. The total imaging time was approximately 6 min. This protocol was repeated twice to increase the signal to noise ratio (SNR).

### Proton MR Spectroscopic Imaging (^1^H–MRS)

A standard PRESS chemical shift imaging ^1^H–MRS was performed with a phase-encoded version of a point-resolved spectroscopy sequence (PRESS) both with and without water pre-saturation as described (Gasparovic et al. 2011). Briefly, the following parameters were used: TE = 40 ms, TR = 1500 ms, slice thickness = 15 mm, FOV = 220 × 220 mm, circular k-space sampling (diameter = 24), total scan time = 2 × 582 s. A TE of 40 ms was chosen to improve detection of the Glx (combined glutamate and glutamine) signal (Mullins et al. 2008). The nominal voxel size was 6.9 × 6.9 × 15 mm^3^ (0.71 cm^3^) but the effective voxel volume is estimated to be 2.4 cm^3^. The ^1^H–MRS volume of interest (VOI) was prescribed from an axial T2-weighted image to lie immediately above the lateral ventricles and parallel to the AC-PC axis, and included portions of the cingulate gyrus and the medial frontal and parietal lobes. To minimize the chemical shift artifact, the transmitter was set to the frequency of the NAA methyl peak during the acquisition of the metabolite spectra and to the frequency of the water peak during the acquisition of the unsuppressed water spectra. Additionally, the outermost rows and columns of the VOI were excluded from analysis. Spectra were automatically fitted with LCModel (version 6.1) in the spectral range between 1.8–4.2 ppm in reference to the non-water-suppressed data using “water-scaling” (Gasparovic et al. 2006), blind to diagnostic group. Only metabolite values with goodness of fit, as measured by the standard-deviation (SD), of ≤20 were further analyzed. Finally, results from LCModel for the metabolites of interest were corrected for partial volume (using SPM-5 segmented T1 images) and relaxation effects (from literature values), as outlined previously (Gasparovic et al. 2011). Metabolites sampled include creatine (Cr), phosphocreatine (PCr), glutamate (Glu), glutamine (Gln), choline (Ch), myo-inositol (Ins), NAA and N-acetylasparthglutamate (NAAG).

### MEG data collection

MEG data were collected in a magnetically shielded room (AK3b, Vacuumschmelze GmbH & Co. KG) using the Elekta Neuromag 306 channel biomagnetometer (VectorView, Elekta AB) located at MRN. Before acquisition of MEG scans, four head-position indicator (HPI) coils were placed around the participant’s head and secured with tape. The HPI coil location and head shape digitization was obtained using the Polhemus 3-D tracking device. Three fiducials (left and right preauricular and nasion) were identified, along with ~200 additional points around the scalp, to define the head-centered coordinate system and for co-registration of the MEG data to the MRI structural image. Two channels of electrooculogram (EOG), vertical (one electrode placed above the left eyebrow and one placed below the left eye) and horizontal (one electrode each was placed lateral to the outer canthus of the left and right eye), were recorded to provide signals for artifact rejection of eye movements and blinks. One cardiac channel (leads placed just below the left and right clavicle) was also recorded simultaneously with the MEG data for identification of heart beat artifacts. Eye-tracking (Eyelink 1000, SR Research) was used during MEG data collection for Project 2 when available (corrective lenses interfere with eye-tracking). MEG data were collected at a sampling rate of 1000 Hz, DC coupled with a low-pass filter at 330 Hz. Continuous MEG raw data were collected in conjunction with continuous head position data (cHPI) for offline correction of head motion using Neuromag’s MaxFilter 2.1 MaxMove. Trials were rejected if magnetic activity was greater than 3000 femtotesla (fT) peak-to-peak in any channel. A subject-specific averaged eyeblink was identified and modeled using the signal space projection method (Uusitalo and Ilmoniemi 1997) within the Minimum Norm Estimates (MNE) Python software (Gramfort et al. 2014). Presentation software (Neurobehavioral Systems) was used for the presentation of stimuli.

### Neuropsychological scales

An extensive neuropsychological battery was performed for each of the participants with neuroimaging data (same as the other projects). The battery of tests administered to SPs included: CPT-IP (Continuous Performance Test-Identical pairs), Quality of Life Scale, MATRICS (Measurement and Treatment Research to Improve Cognition in Schizophrenia), WASI (Wechsler Abbreviated Scale of Intelligence), WAIS-IV (Processing Speed Index of the Wechsler Adult Intelligence Scale-IV which includes symbol search and coding), WTAR (Wechsler Test of Adult Reading), and UCSD Performance-based Skills Assessment (UPSA/Psychosocial Functioning). HCs were administered the following tests: CPT-IP, MATRICS, WASI, WAIS-IV (symbol search, coding PSI) and WTAR.

### Genetic analyses

In addition, our COBRE I studies included the collection of genetic material from subjects to evaluate the genetic contributions to neuroimaging findings in all the four projects and a pilot project. A number of genes have been associated with increased vulnerability for schizophrenia (Ripke et al. 2013). This genetic information adds a new and important dimension for the interpretation of endophenotypes revealed by neuroimaging studies. Saliva was collected and DNA was isolated and analyzed using genome wide genotyping chips. Saliva was collected from 100 SP and 100 HC subjects. Of these, 84 samples were genotyped using Illumina’s HumanOmni Quad 1 M Beadchips (Illumina, San Diego) spanning 1,140,419 SNPs and the rest were genotyped using Illumina’s HumanOmni Quad 5 M beadchips containing about 5 million SNPs and two samples were genotyped in both arrays. GenomeStudio software was used to make the final genotype calls. A series of quality control procedures following the recommendation by Anderson et al. (2010) was performed in PLINK (Purcell et al. 2007), including less than 5% per-sample missingness, samples’ gender match, heterozygosity within 3 SD, relatedness <0.18 (no 2nd degree or closer relatives), Hardy-Weinberg equilibrium <1 × 10^−6^, and minor allele frequency (MAF) > 0.05 (Chen et al. 2012). Although the vast majority of subjects self-reported racial status, we validated this information by computing the population structure from the genotyping data. Specifically, we applied PLINK multidimensional scaling analysis to our samples and used the HapMap3 as the reference. In addition, we used TaqMan® genotyping probes to assay another SNP not present in the 1 M chip, the rs1625579 in the miR-137 host gene.

### Description of Tasks and Data Acquired

#### Project 1

The first phase of Project 1 focused on basic pre-attentional (Task 1) and attentional processes (Task 2), whereas the second phase of the project focused on multisensory cognitive control (Task 4) and detection (Task 5). Task 3 was common to both phases of the project and involved a resting state fMRI scan. All fMRI/MRI data were collected on a 3.0 Tesla Siemens Trio scanner using a 12-channel head coil as described previously under the “MRI/fMRI data collection” section. MEG data were acquired for two of the tasks using an Elekta Neuromag 306 channel system as described under the “MEG data collection” section. See Table 1 for the number of SPs and HCs participating in each task. MEG task parameters are described below.

##### Project 1 Tasks 1 and 2

For Task 1 hemodynamic response functions were explicitly modeled for single (S1) and pairs (S1 + S2) of identical (IT; “gating-out” redundant information) or non-identical (NT; “gating-in” novel information) tones (Mayer et al. 2013). For Task 2, cues presented to the left or right ear (100 ms, 2000 Hz tone pip) correctly predicted the location of the targets (100 ms, 1000 Hz tone pip) in 50% of the trials (Abbott et al. 2012). Participants pressed a button with their right middle finger for tones on the right headphone and a button with their right index finger for tones on their left. The stimulus onset asynchrony (SOA) between cue and target was 200, 400, 600 or 800 ms, which was designed to effectively capture both facilitation (200 ms SOA) and inhibition of return or IOR (800 ms), as well as approximate the crossing of the two functions (400 and 600 ms SOA). For Task 2 the onset of a cue and the onset of the next cue ranged from 3 to 5 s at multiples of 1 s, with 240 trials acquired for each ISI (interstimulus interval). Cue and Target durations were 100 ms. The majority of participants who completed Tasks 1 and 2 also completed a resting state fMRI scan (Task 3), in which participants passively stared at a fixation cross for approximately 5 min. An MEG resting state scan (Task 3) utilized the same parameters as for the fMRI rest condition. A variant of Tasks 2 and 3 was also collected using MEG.

##### Project 1 Tasks 4 and 5

**Fig. 2:**
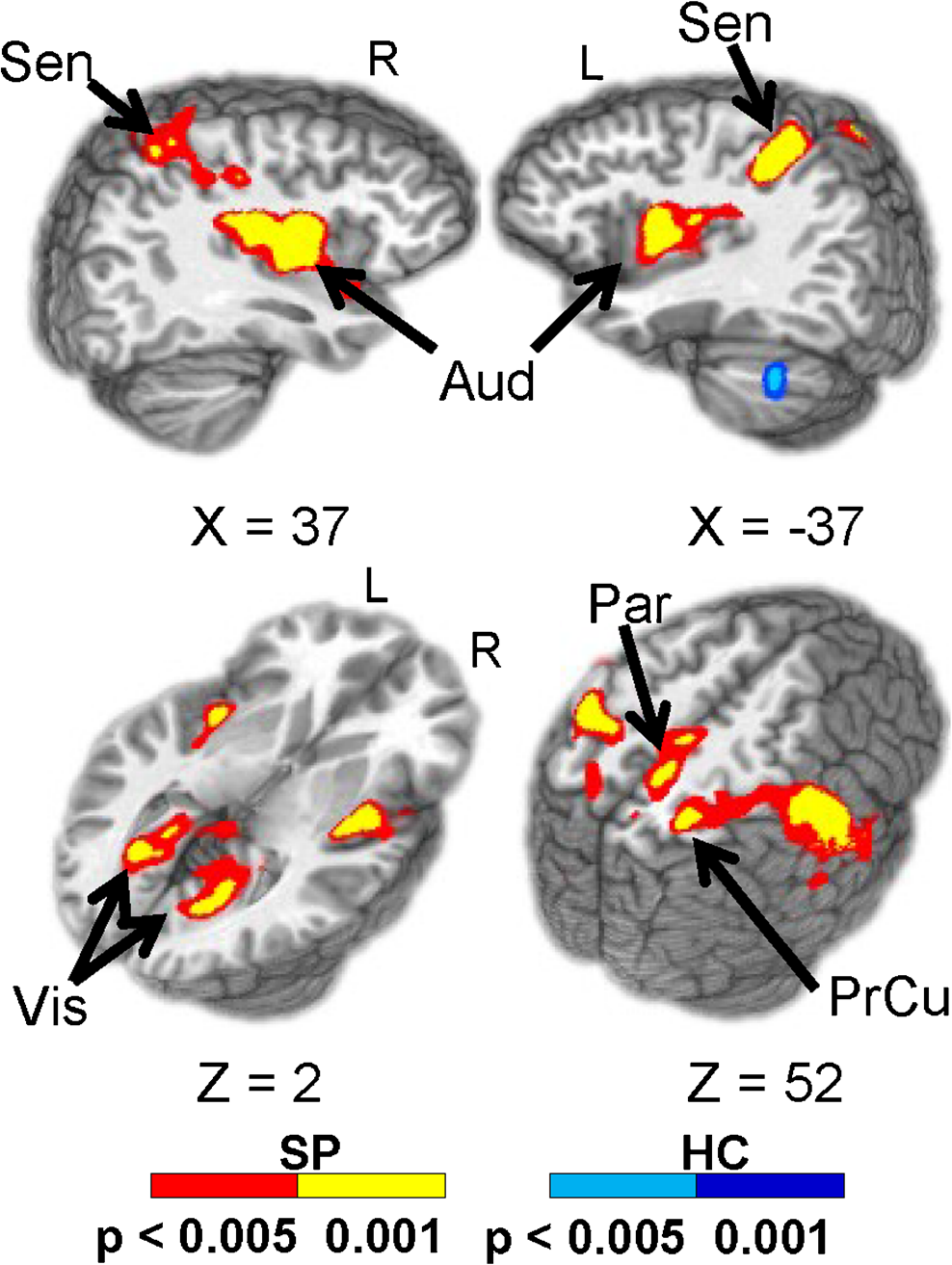
Brain regions showing significant group differences between SP (warm colors) and HC (cool colors) during the attend-visual (AV) condition. Locations of the sagittal (X) and axial (Z) slices are given according to the Talairach atlas (Talairach and Tournoux 1988) for the left (L) and right (R) hemispheres. Adapted from Mayer et al. 2015

In Task 4, congruent or incongruent multisensory (auditory and visual) numeric stimuli were simultaneously presented at either low (0.33 Hz; 3 trials/block) or high (0.66 Hz; 6 trials/block) frequency rates in ten-second blocks (Mayer et al. 2015). In each block, the stream of target numbers (one, two, or three) was preceded by a cue word, “HEAR” or “LOOK”. If the cue was “HEAR,” participants were instructed to respond via a right-handed button press to aurally presented target stimuli and ignore simultaneously presented visual numbers (attend-auditory condition). When the cue was “LOOK,” visually presented stimuli were the targets and participants were instructed to ignore auditory stimuli (attend-visual condition). In addition, the task also contained a proactive response inhibition condition in which participants were asked to refrain (cue word = “NONE”) from making a button press (Mayer et al. 2016). Results from this task are shown in Fig. 2. In general, SP revealed an overall pattern of response slowing which was associated with a pattern of patient hyperactivation within auditory, sensorimotor and posterior parietal cortex, compared to HC. However, there were no group differences in functional activation within prefrontal nodes of the CCN (Mayer et al. 2015). Finally, Task 5 used a multisensory detection task with a rapid event-related fMRI paradigm to investigate whether both the positive and post-stimulus undershoot of the hemodynamic response function are affected in SP. For this task, the target stimuli consisted of a simultaneously presented blue box (visual angle = 4.3 × 6.5^○^) and auditory tone (2000 Hz re-sampled with a 10 ms linear ramp) for 300 ms duration. Participants responded with a button press upon the detection of the stimuli (Hanlon et al. 2016).

#### Project 2

##### Forced Choice Multisensory Auditory/Visual Task [see (Stone et al. 2011) for a complete description]

The task for Project 2 was designed to investigate cortical disconnection in SP using fMRI and MEG measures from an auditory and visual multisensory task (EEG data were acquired simultaneously for a subset of SP and HC—these data are available upon request). The following 8 conditions were presented in random order to each participant: auditory alone (near/far = cond1/cond2), visual alone (near/far = cond3/cond4), synchronous auditory and visual (AV: near/far = cond5/cond6) and asynchronous AV (near/far = cond7/cond8). The near/far manipulation represented two different volume levels for the auditory stimuli (loud = near, quiet = far) and two different locations within the visual field with a soccer ball presented in a soccer field background (near = peripheral, far = foveal). The image of the black and white soccer ball appeared on the static soccer field background for 200 ms. For the V Near condition, the ball was centered at 8 degrees below fixation and subtended 2.7 degrees visual angle. For the V Far condition the soccer ball appeared at 1.8 degrees below fixation and subtended 1 degree of visual angle. For the auditory Near and Far conditions a 550 Hz 200 ms duration tone was presented at 63 and 45 dB SPL, respectively. The same stimuli were used for the multisensory conditions. In all cases the conditions were congruent (e.g., Near ball/Near tone). During 20% of the trials, feedback indicating a correct or incorrect response was provided to participants. The feedback was consistent with the trial condition (auditory, visual or combined) and consisted of either a crowd cheer and the soccer ball rolling into the goal for correct responses or a crowd groan and the soccer ball missing the goal for incorrect responses. For the MEG study, the ISI was randomized between 1500 and 2000 ms and all 8 conditions were randomized and presented in 6 separate blocks. Each participant was presented with 150 trials of each condition resulting in ~140 trials per condition after artifact rejections. An auditory threshold test was performed in the scanner prior to data collection to normalize the sound volume across participants. Please see Fig. 1 in (Stephen et al. 2013) or (Stone et al. 2014) for examples of the visual display used for data acquisition. Synchronous AV stimuli had a 5 ms lag between the presentation of the auditory and visual stimuli (with visual presented prior to auditory), whereas for the asynchronous AV condition the auditory stimulus lagged the visual stimulus by 50 ms (representing the natural delay experienced when a sound source is located approximately 50 ft. from the listener). The task was a simple forced choice task that required the participant to respond to all 8 randomized conditions by deciding whether the stimulus was “near” or “far” governed by the conditions stated above. As noted in Table 1, approximately 67 HC and 68 SP participated in each of the tasks during fMRI and MEG scanning.

Behavioral data were collected for both the fMRI and MEG tasks. Individual trial reaction times (RTs) and correct/incorrect responses were recorded. For fMRI data acquisition, 80 averages were acquired for each condition. An ORASI rest paradigm was used for resting MEG acquisition which consisted of 3 min eyes closed, 1 min eyes open, with instructions to blink at the beginning to allow for good characterization of eye blinks during the eyes open portion of the rest task. Raw and Maxfiltered MEG data (.fif format) are available. DICOM and preprocessed MRI data (through MRN’s SPM pipeline) are also available.

**Fig. 3:**
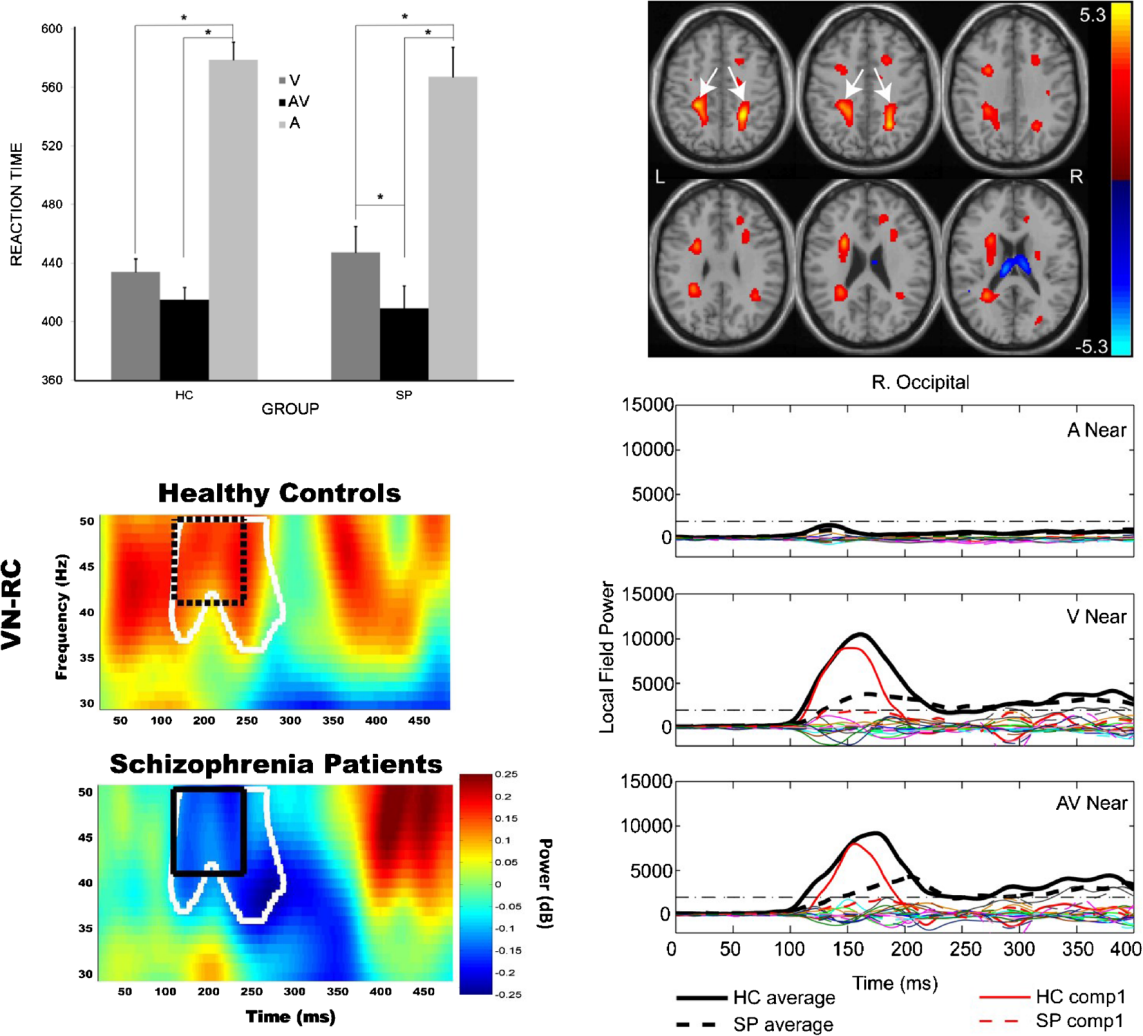
Upper left panel: Reaction times (RTs) to auditory, visual and AV stimuli in SP relative to HC from Stone et al. (2014). Lower left panel: Example of differences in gamma band power in SP vs. HC time-frequency maps. Frequency is represented on the y axis while time in ms is represented on the x axis. In this example gamma band power was greater in HC relative to SP (see black box). Upper right panel: Joint ICA component of fractional anisotropy (FA) consistent with the superior longitudinal fasciculus showed larger FA in HC vs. SP. Similarly, the MEG signal over occipital cortex was significantly greater in HC (red solid line) vs. SP (red dashed line). The joint component weighting factor positively predicted performance on the MATRICS cognitive battery suggesting that visual processing and structures that link visual cortex to frontal regions are important for cognitive performance

Example results of this study are shown in Fig. 3. Contrary to our initial hypothesis, SP did not show impaired multisensory integration. This is illustrated in behavioral results shown in Fig. 3A. Behavioral multisensory benefit is relative to unisensory responses. In this case, the RTs in the AV condition were statistically equivalent for HC relative to the visual RTs. This shows that multisensory information did not lead to a significant facilitation of the RTs for HC. However, for SP the RTs were significantly faster for the AV relative to the fastest unisensory condition (V). Although the V RTs were significantly different by group, the AV RTs did not differ by group suggesting that the addition of the auditory stimulus helped to normalize the behavioral response in the SP group. The results in Stone et al. (2011) also showed that the electrophysiological response was similarly normalized (unisensory response was lower amplitude in SP vs. HC, whereas the multisensory response was not significantly different by group). These behavioral results were replicated in the full COBRE dataset (Stone et al. 2014). Gamma oscillations have also been implicated in feature binding. As a follow-up to our results (Stone et al. 2011), we investigated whether differences in gamma band oscillations could help explain the behavioral and physiological differences in multisensory processing in SP. Our results indicated that group differences in gamma band oscillations were observed in both posterior and frontal regions, yet there was not a direct relationship to multisensory behavioral benefit. Our interpretation is that gamma band oscillations may play a partial role in multisensory processing, but it is likely that additional oscillations and network connectivity is also an important component of multisensory integration in SP. Finally, a primary goal of the COBRE project was to integrate data across modalities. The joint ICA approach developed by the COBRE PI, V. Calhoun, was applied to MEG and dMRI data (Stephen et al. 2013). Our motivation for combining these modalities is that multisensory integration requires precise timing of information between unisensory and polysensory areas and MEG provides high temporal sensitivity to regional activation whereas dMRI allows one to assess the WM integrity underlying the connections between these regions. Our results indicate that variations in MEG amplitude/timing were directly associated with alterations in WM integrity in SP versus HC. These results provide evidence for alterations in structure that are associated with alterations in function as measured by MEG. These linkages across modalities may provide a better understanding of the structure/function networks that underlie the cognitive and social impairments experienced by individuals with schizophrenia.

#### Project 3

##### TP and Elemental Tasks [for a complete description of methods see (Hanlon et al. 2012)]

**Fig. 4:**
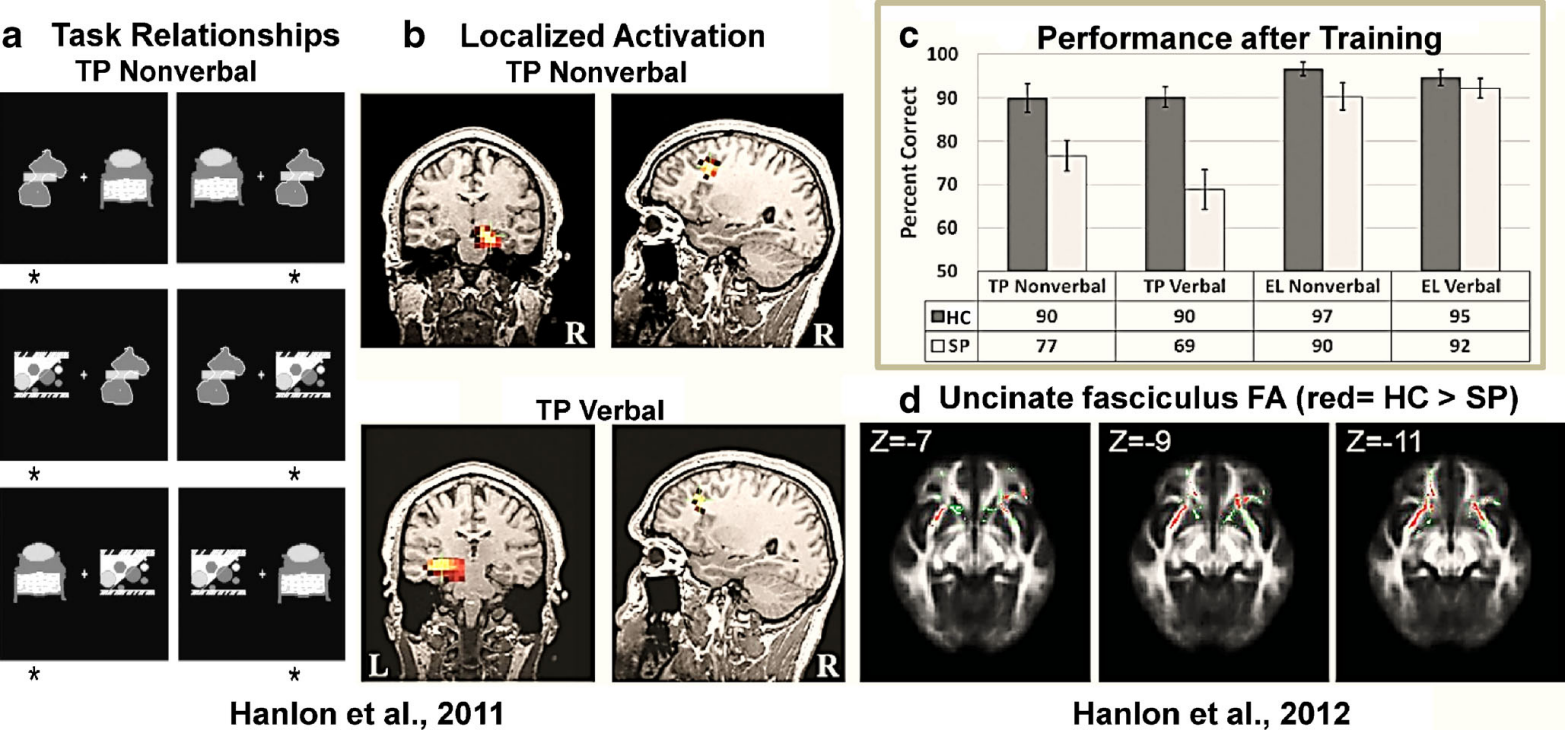
**a**. Project 3 task relationships for the nonverbal TP task. The 6 images represent the 6 possible stimulus pairings per trial. Each pairing is presented randomly and sequentially to the participant. The participant was asked to choose the correct stimulus in each pairing using a mouse button press (asterisks indicate the stimulus choice). **b.** Hippocampal and PFC activation from the sLORETA analysis, plotted on one HC’s MRI for each version of TP. These images show the most common activation pattern for HC. **c.** Performance results for nonverbal and verbal versions of the TP and Elemental (EL; control) tasks. **d.** Red tracts indicate where FA values were lower for SP compared to HC in the uncinate fasciculus (green = the uncinate fasciculus skeleton)

Participants were trained on the verbal and nonverbal versions of the TP task (Tasks 2 and 4) and their elemental counterparts (Tasks 1 and 3) to a criterion of 18 consecutive correct responses in a row, or 200 trials if criterion was not met, on each task. Presentation stimulus software version 13.1 (Neurobehavioral Systems) was used to present stimuli. Stimuli for the nonverbal versions of the TP and elemental tasks consisted of three different abstract black and white pictures, unique in appearance for each of the tasks (see Figure 4A). Stimuli for the verbal versions of the TP and elemental tasks consisted of three pronounceable non-words for each task. On each trial, two stimuli were presented simultaneously, one centered on the left side of the screen and one centered on the right, and the participant chose which of the two stimuli was correct via a button press. For the TP task versions, stimuli A, B, and C were used (Fig. 1 in Hanlon et al. 2012). A is correct when presented with B. B is correct when presented with C. C is correct when presented with A. For the elemental task versions, stimulus D is correct when presented with E or F. Stimulus E is correct when presented with F. Each pair of stimuli was presented until the participant responded or until 3 s had passed for all tasks. The response was followed by feedback, a high-pitched tone if correct and a low-pitched tone if not correct. There was a randomly jittered 3 to 5 s inter-trial interval (ITI) from button press or end of the 3 s window until the next stimulus onset, during which a fixation cross appeared in the middle of the screen. The presentation, task order, and instructions for the tasks were the same for training, MEG scans, and fMRI scans, except that the fMRI scans had a longer jittered ITI of 8 to 12 s to facilitate sampling of the hemodynamic response (Burock et al. 1998). Approximately 60 HC and 47 SP participated in each task during fMRI and MEG scanning (Table 1).

Across our studies using MEG and the verbal and nonverbal versions of the TP task, we have found lateralized hippocampal and PFC activation deficits in SP, as well as lower fronto-temporal anatomical connectivity which is related to working-relational memory performance deficits and worse every day functioning in SP (Hanlon et al. 2012, 2011, 2003, 2005b). Specifically, Hanlon et al. (2011) found that SP exhibited lower mean behavioral performance than HC on both the nonverbal and verbal version of TP in the MEG, with no decrement in performance on the non-hippocampal-dependent elemental (EL) control task versions. Fig. 4B displays an example of the hippocampal and PFC activation (shown in color) found in a HC using the standardized Low Resolution Brain Electromagnetic Tomography (sLORETA) analysis (Pascual-Marqui 2002, Wagner et al. 2004). HC showed more right hippocampal activation during nonverbal TP and more left hippocampal activation during verbal TP (Hanlon et al. 2011). This lateralized hippocampal activation was not seen in patients, who instead showed more bilateral or left hippocampal activation for both TP versions. We also found that SP exhibited more left PFC activation (BA 9 and 10) while HC exhibited more right PFC activation for both versions of TP (Fig. 4B). In addition, using COBRE data we examined the relationship between fronto-temporal anatomical connectivity with working/relational memory performance (nonverbal and verbal versions of TP) and everyday functioning (Hanlon et al. 2012). Fronto-temporal anatomical connectivity was assessed using dMRI measures of FA in the uncinate fasciculus. The tract based spatial statistics (TBSS/FSL) method was used to calculate participant specific skeletons (Smith et al. 2006). The skeleton was further restricted to uncinate fasciculus as defined by the JHU atlas (Wakana et al. 2004). Group differences were evaluated by comparing group mean values for FA over the uncinate fasciculus skeleton (Fig. 4D; green voxels; MNI z-coordinate indicated). The UPSA-2 (Patterson et al. 2001) was included to evaluate everyday functioning in patients. Results again showed that patients performed worse than HC on both the verbal and nonverbal versions of the TP task, but did not show a performance decrement on the verbal or nonverbal versions of the elemental (EL) control task (Fig. 4C). Also, FA in bilateral uncinate fasciculus was lower in SP compared to HC (Fig. 4D; red voxels). Finally, lower fronto-temporal anatomical connectivity (lower FA) was related to lower working-relational memory performance, and both predicted worse every day functioning (Hanlon et al. 2012).

#### Project 4

The analysis of the P-FIT model of creativity has been studied in healthy individuals (Vakhtin et al. 2014) but has not yet been evaluated in the schizophrenia data. Multiple studies, however, using structural connectivity, ^1^H–MRS, and sMRI data, have been published as discussed below. Each COBRE investigator utilized structural and biochemical data acquired under Project 4 to enhance their existing projects (i.e., converging multimodal data); therefore, the focus here is on descriptions of the structural and ^1^H–MRS data that is available to others in terms of raw and preprocessed data.

##### Diffusion Tensor Imaging (DTI)

A DTI processing pipeline has been developed that was used by several studies at MRN that needed DTI analysis (Aine et al. 2011, Caprihan et al. 2011, 2015, Hanlon et al. 2012, Monnig et al. 2013, Wu et al. 2015) and by other research groups outside of MRN (Bessette et al. 2014, Haney-Caron et al. 2014). The preprocessing is primarily based on FSL software with some custom MATLAB programs for quality control. The preprocessing steps consisted of data quality check, motion and eddy current distortion correction, and correcting diffusion gradient directions (Caprihan et al. 2011). The output of the preprocessing step is then used for calculating scalar diffusion parameters, TBSS based analysis, source based morphometry (SBM), and structural connectivity analysis. The group studies were based on differences observed in tracts defined by an atlas (Aine et al. 2011), differences based on the skeleton as defined by the TBSS (Caprihan et al. 2015), a data driven method based on SBM of diffusion data to look for group differences in the loading coefficients (Caprihan et al. 2011), and whole brain connectivity matrix differences (Wu et al. 2015). The preprocessed data of subjects scanned under the COBRE project is available for other users through COINS.

##### Proton MR Spectroscopic Imaging (^1^H–MRS)

**Fig. 5:**
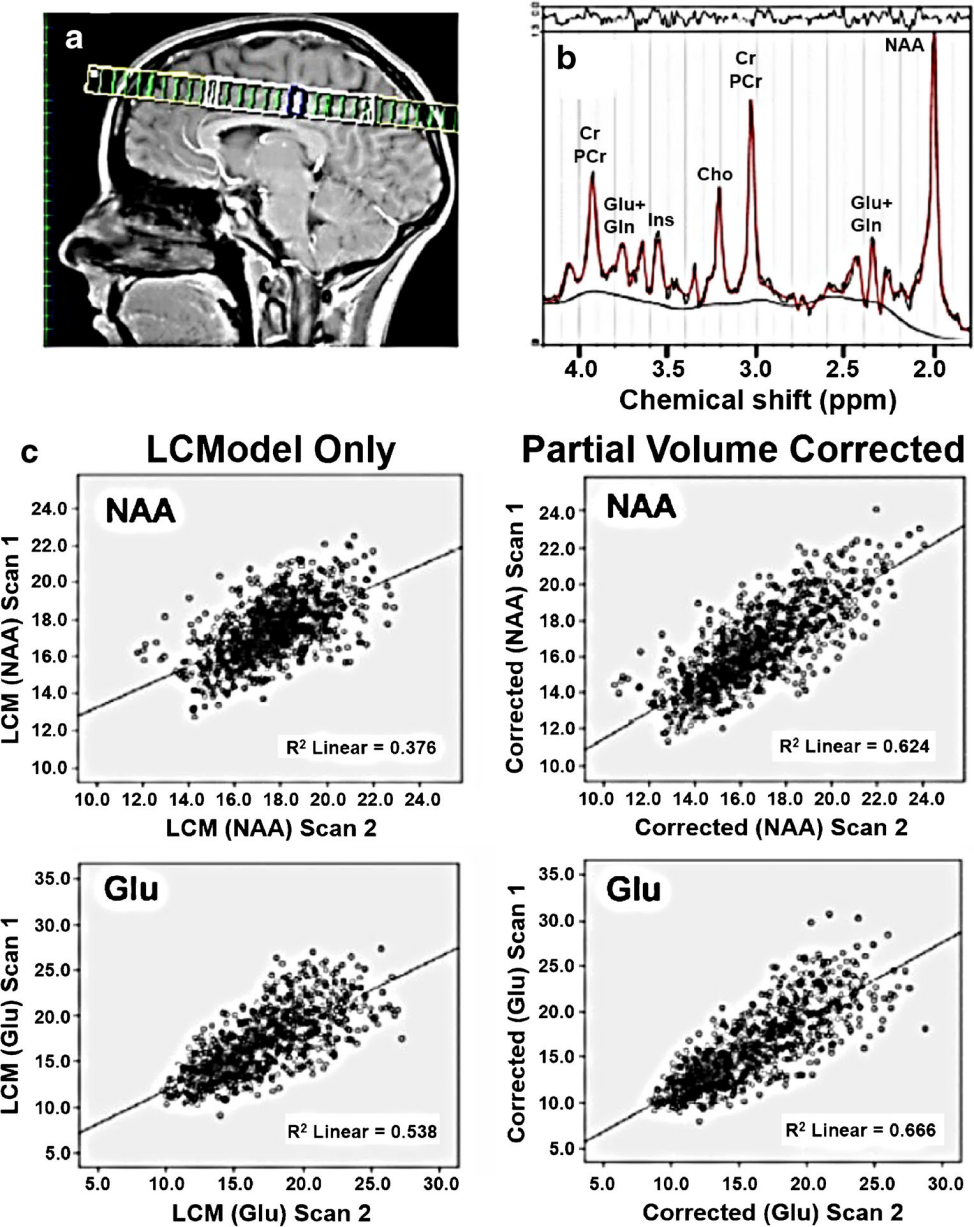
**a.** ^1^HMRS slice location. **b.** A representative fit by LCModel to a spectrum from a voxel within primarily gray matter. **C.** Plots of first scan vs. second scan data for NAA and Glu for all voxels from all subjects with and without partial volume and relaxation correction, respectively. Adapted from Gasparovic et al. 2011

We have ^1^H–MRS data analyzed for 156 subjects and the final auto-analysis results provide for each analyzed voxel the LCModel estimated concentrations and partial volume corrected concentrations of: total creatine (Cr + PCr), Glu, Gln, combined Glu and Gln (Glx), choline (primarily glycerophosphorylcholine and phosphorlycholine; GPC + PCh), Ins, and combined NAA and N-acetylasparthglutamate (NAA + NAAG). The result also captures partial volumes in the spectroscopic voxel and the estimation uncertainty. An example of ^1^H–MRS data is shown in Fig. 5 where test-retest reliability and reproducibility were examined for short-echo time at 3 T [see (Gasparovic et al. 2011) for details]. In this example, measurements of Glu, Glx, Ins, NAA, creatine and choline were acquired in 21 healthy subjects using a 2-dimensional spectroscopic imaging version of a PRESS sequence prescribed above the lateral ventricles. A single excitation slice was prescribed above the lateral ventricles as shown in Fig. 5A and repeat scans were taken within 30 min. All measures were calculated with and without partial volume correction (left and right columns of Fig. 5C, respectively). Overall these results show high reproducibility and test-retest reliability of tissue-specific estimates of several metabolites using ^1^H–MRS at 3 T and short TE in a group of healthy subjects. In addition, a data-driven approach to analyze ^1^H–MRS data using ICA was developed (Kalyanam et al. 2013, 2015). We are in the process of applying this approach to the spectroscopy data collected from controls and schizophrenia patients.

##### Structural imaging

The structural T1-weighted images are freely available, and have been included with other datasets in the analysis of GM covariation patterns in schizophrenia (Gupta et al. 2015) and a world-wide meta-analysis of subcortical volumes in schizophrenia (van Erp et al. 2016). We have also combined the healthy controls from the COBRE study with multiple other data sets collected at MRN in order to evaluate the relationship between structural patterns of covariation and resting fMRI networks (Segall et al. 2012). The structural MRI from project 4 and functional MRI data from project 1 were also utilized in a schizophrenia classification challenge hosted on the kaggle.com competition site and garnered over 2000 entries [(Silva et al. 2014) more details are provided in the section below].

### Sample Data-Driven Analyses of COBRE Data

In this section we describe three more recent endeavors using these data sets: 1) analysis of multi-task fMRI data across projects; 2) use of these data sets for an international signal processing competititon; and 3) examination of functional network connectivity (i.e., network structure and network dynamics) using resting state fMRI and MEG in SP and HC.

#### Multi-Task Analysis of fMRI Data across Project Tasks

**Fig. 6:**
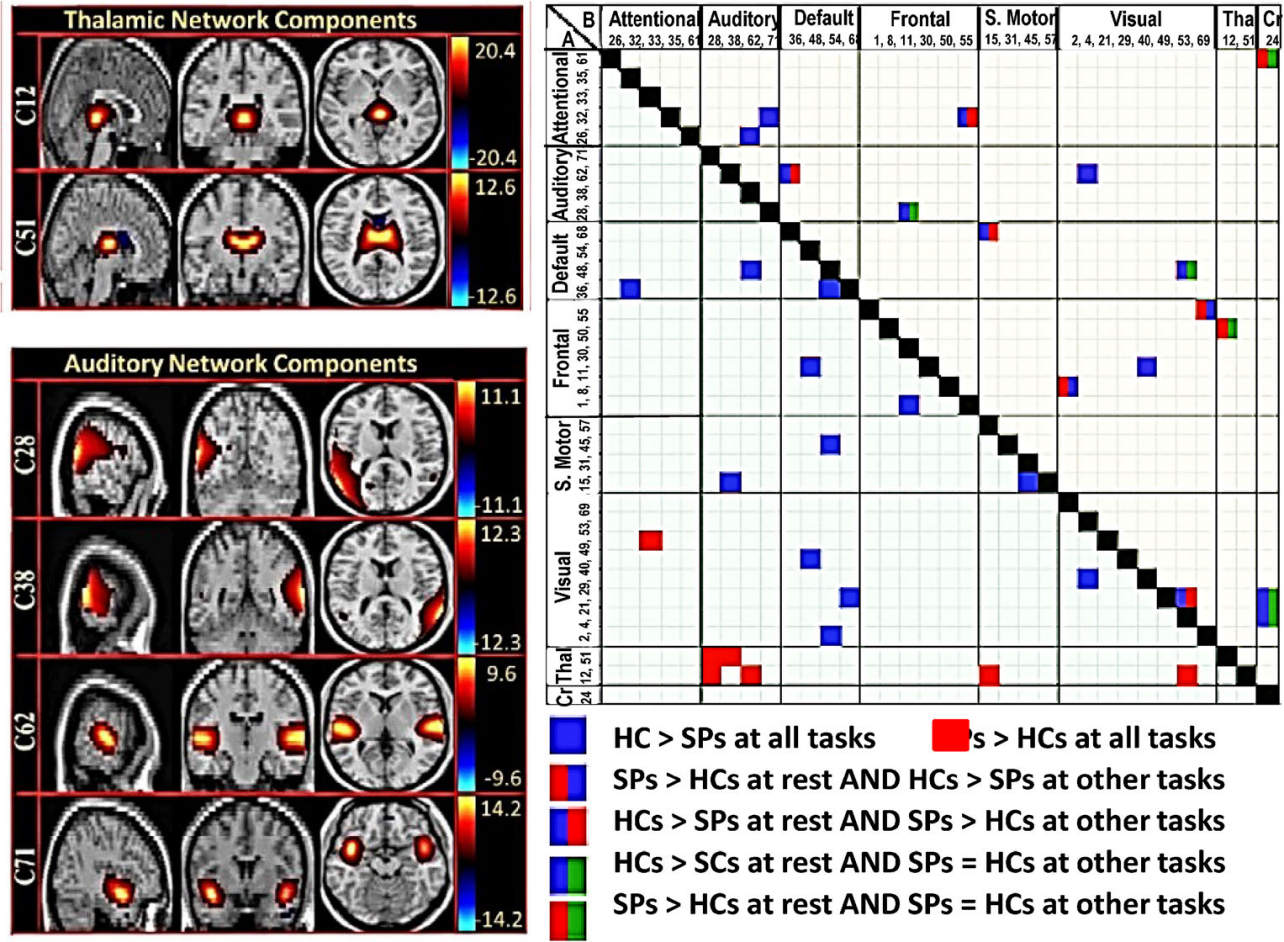
**Left panel**. Maps of the components identified as non-artifactual in static FNC or dynamic FNC analysis: Of the 75 components returned by the GICA, 45 were identified as non-artifactual components. Only 34 of these non-artifactual components showed static FNC or dynamic FNC effects; these 34 components were divided into groups based on their anatomical and functional properties and include visual network, thalamic network, cerebellar network, frontal network, attentional network, default mode network, sensory motor network, and auditory networks. Only 6 components, representing the thalamic (C12, C51) and auditory networks (C28, C38, C62, C71) are shown. Color bars at the right represent z-scores. **Right panel. a.** Static FNC matrix (lower part). Pairwise correlations of component pairs showed static FNC effects at α > 0.001 level. **b.** Dynamic FNC matrix (upper part). Pairwise correlations of component pairs showed dynamic FNC effects at the α ≤ 0.001 level. Thal = thalamus network, CR = Cerebellar network. Adapted from Cetin et al. 2014

We analyzed fMRI data from 53 HC and 42 SP obtained during rest and selected tasks from each project [for further information see (Cetin et al. 2014)]. Data for each participant were gathered across multiple fMRI scanning sessions over the course of up to 2 months (1–2 months) with prospective randomization of task presentation and close monitoring of SPs to ensure clinical stability. Functional network connectivity (FNC) based on group ICA results computed from all fMRI tasks plus rest fMRI was assessed with different cognitive demands: resting state, pre-attentive sensory processing and multisnesory processing. We used a novel analysis for assessing across paradigm connectivity patterns to identify stable (static effects) and state-based (dynamic effects) differences between HC and SP groups. A group by task full factorial ANOVA model was applied to the group average network connectivity values. Figure 6 (left panel) shows 6 of the 45 non-artifactual brain networks identified using a group independent component analysis (GICA). The right portion of the figure shows between-group differences in static FNC (across all tasks; A) and dynamic FNC (only for some tasks; B); *increased* static FNC in SPs was observed in a network that included the thalamus in addition to auditory, motor and visual cortices (see cluster of red boxes in lower left portion) consistent with a trait deficit in thalamic filtering. However, *reductions* in static FNC between the default mode network and frontal, motor and visaul cortices were also present in SP relative to HC. Finally, dynamic FNC differed in more complex ways; for example, SPs showed significantly higher FNC than HCs in resting state in the thalamic/frontal network pair, while HC FNC was higher during performance of the task-related paradigms. These results suggest that SP network connectivity deficits change depending on whether the participant is resting or actively engaged in a task, and furthermore these connectivity patterns change across task hierarchy. This approach provides a clear advantage in recognizing the complex variability in brain reactions to simple sensory stimuli, which are conditioned by experimental circumstances, in SP and HC populations. Similar integration of data across tasks and/or other imaging modalities will be supported for future external users.

#### Tenth Annual MLSP Competition—Schizophrenia Classification Challenge

**Fig. 7:**
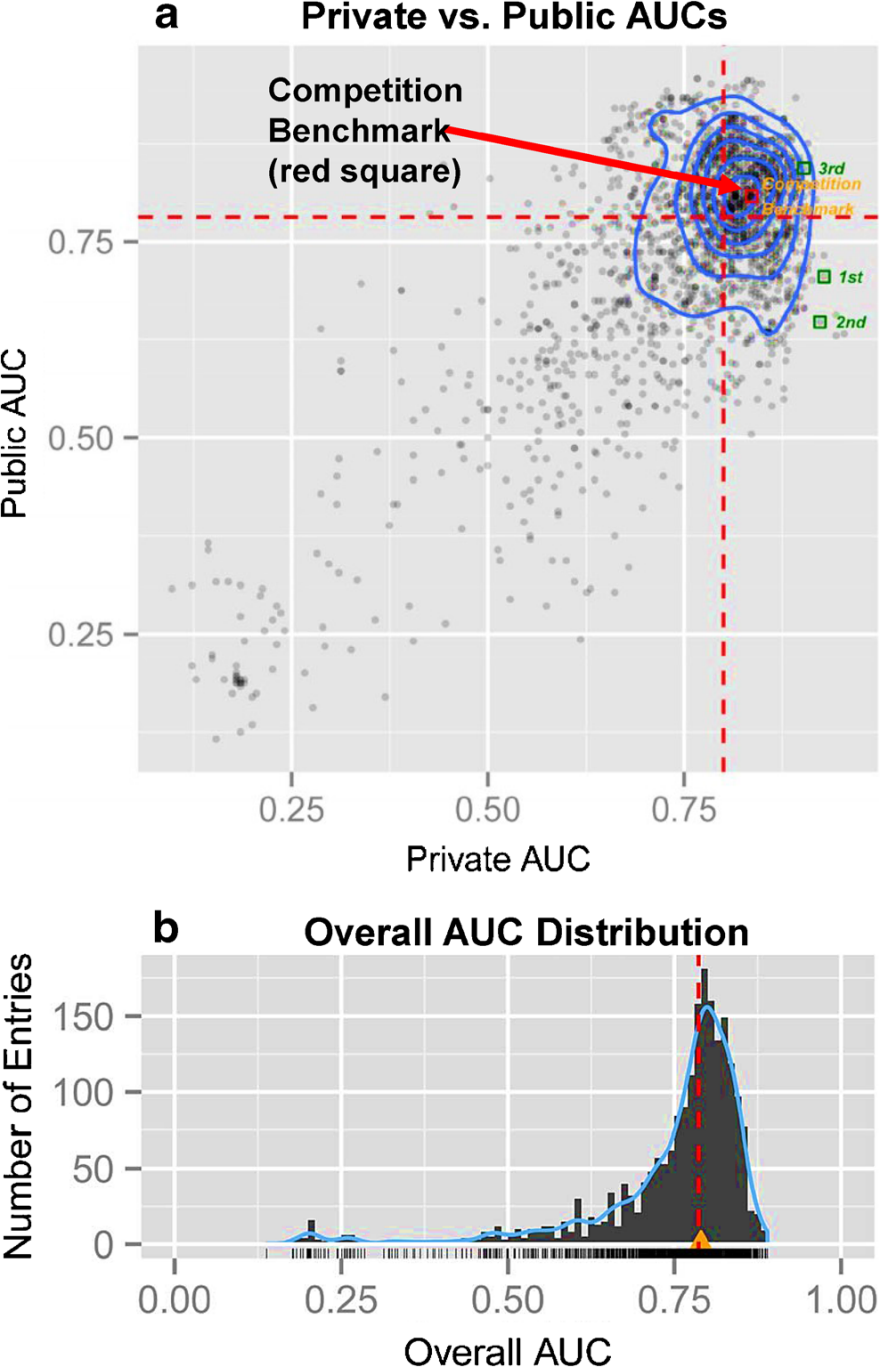
**a.** Dashed red lines represent median values of Public and Private AUCs. Blue contours indicate the centration of entries. The 3 winning entries are shown as green squares at the upper right. 1st and 2nd place entries attained lower public AUCs because the official ranking optimized for extremes along the x-axis only. **b.** Overall AUC of all 2087 entries. Dashed red line indicates the median. Orange/yellow triangle indicates the competition benchmark (just above the median). No entry was able to attain an overall AUC of 0.9 or higher. Adapted from Silva et al. 2014

The 10th annual Machine Learning for Signal Processing (MLSP) competition utlized a portion of the COBRE dataset of HCs and SPs with the goal of identifying the best objective classification of schizophrenia patients based on multimodal features derived from MRI scans. Each investigator (291 investigators and 2087 entries) developed a classifier, optional feature selection, that combined structural and functional MRI features, hence providing an example of multimodal data fusion (Calhoun and Sui 2016). The test sets were divided into Public (30 subjects) vs. Private (28 subjects) sets; multiple entries were permitted with feedback provided for Public test sets only (area under the curves or AUCs). Because there was variability between the Public and Private AUCs (Fig. 7 top), most likely due to the small sizes of the data sets, the overall distribution of overall AUCs of the entire test set was used instead. Figure 7 (bottom) shows the overall AUC distribution for all 2087 entries; many entries were able to attain an overall AUC of 0.8 or higher and several different strategies produced good results. For example, the team winning first place estimated class probabilities for test examples by means of a Gaussian process classifier with prior distribution scaled by a probit transformation. For more information on the winning strategies and the competition itself please see (Silva et al. 2014).

#### Functional Network Connectivity in both fMRI and MEG

**Fig. 8:**
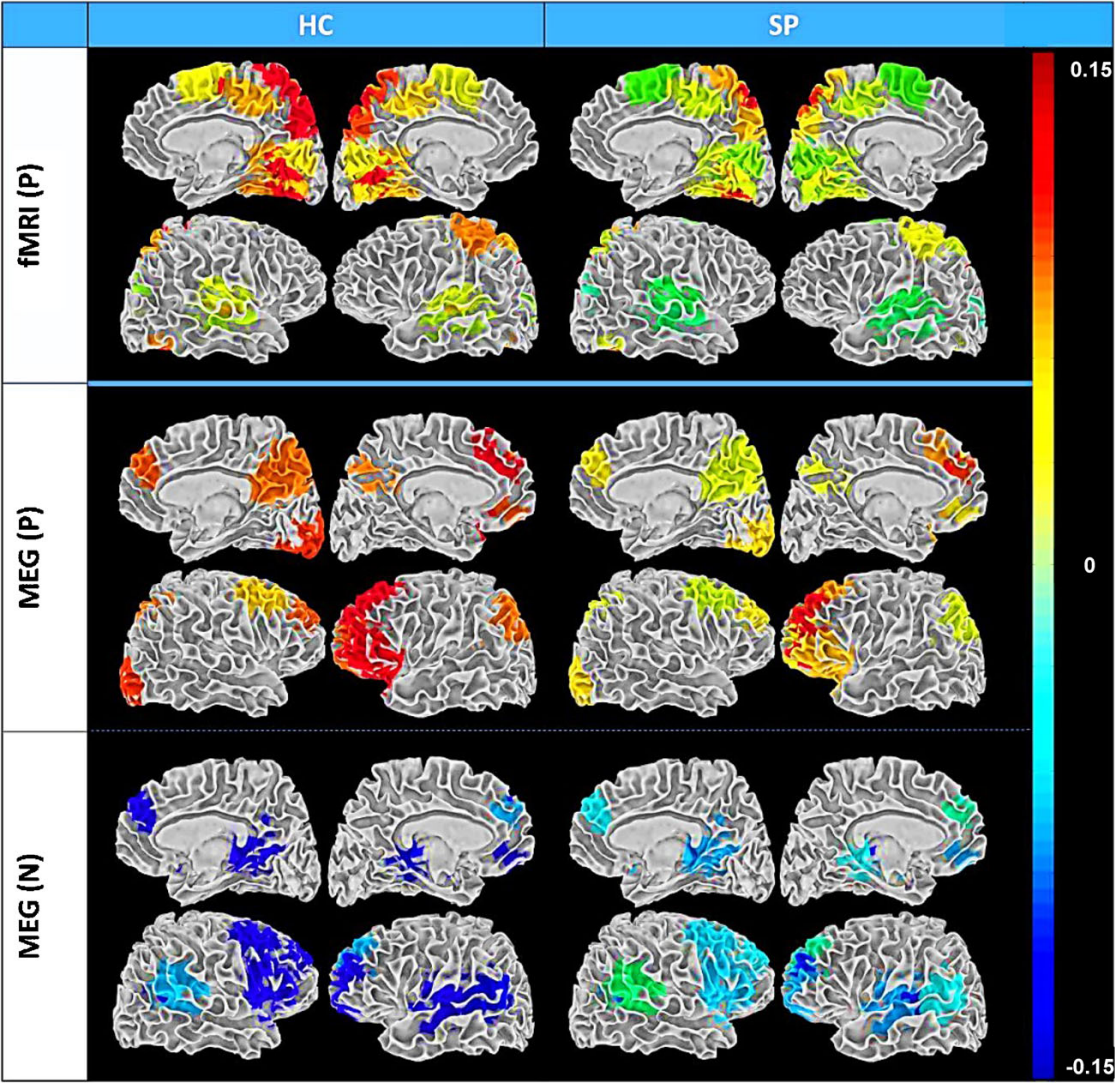
Summary of FNC group averages (SP and HC) for fMRI and MEG rendered on white matter surface. P = HC > SP and N = SP > HC. FNC is a measure of among- network connectivity; that is, pairwise correlations in network (ICA component) timecourses. Only those regions involved in significant group differences are included. Color bar values are the mean correlations for each component and group. Importantly, the MEG and fMRI results were quite different from one another (i.e., greater FNC was observed in HC visual networks for fMRI components while greater FNC was evident in SP frontal networks for MEG components (blue color), highlighting the complementary information embedded within these two modalities. We adjusted for multiple comparisons within each network matrix using the false discovery rate correction. Adapted from Houck et al. 2017

Network structure and network dynamics (functional network connectivity or FNC) were examined using resting state fMRI and MEG data obtained from 46 SP and 45 HC (Houck et al. 2017). Our resting fMRI FNC findings are consistent with work indicating hypoactivation in prefrontal cortex (Kuhn and Gallinat 2013). In contrast, prefrontal FNC was enhanced (i.e., hyperconnectivity) in SP for MEG (see bottom panel of Fig. 8--blue), suggesting abnormally high synchronous firing from neuronal populations in prefrontal and temporal networks. It is not possible to determine with certainty from these results whether these hyper-synchronous networks underlie core deficits of the illness, or whether they represent compensation to overcome primary functional defects. However, our MEG results suggesting hyper-synchronous prefrontal and temporal networks are consistent with the dysconnectivity model of schizophrenia (Stephan et al. 2006). Both similar and contrasting patterns of connectivity were identified in fMRI vs. MEG indicating that these modalities which measure brain function at different time scales using fundamentally different measures (hemodynamic vs. electromagnetic activity) provide access to complementary information. This point is reiterated in a separate analysis using the resting state connectivity data to determine classification accuracy with fMRI alone, MEG alone, and combined fMRI and MEG data (Cetin et al. 2016). By using both static and dynamic connectivity measures, the correct classification rate was highest using the combined fMRI and MEG connectivity measures.

These datasets also provide a unique opportunity for fusing data of various types, besides combining data across modalities. The first study mentioned in this section (Cetin et al. 2014) can be considered as multi-task data fusion and the Silva et al. (2014) competition challenge paper requires data fusion across sMRI/fMRI. Stephen et al. (2013) and Sui et al. (2015) provide more examples of the richness of these datasets where MEG data is fused with DTI data, and where MATRICS responses are fused with fMRI/dMRI/sMRI.

### Final remarks

Neuroimaging studies of schizophrenia have typically identified small differences between SP and HC in electrophysiological, structural, functional and metabolic brain measures. However, many of the functional/structural studies have focused on single task measures (or resting state), unisensory measures, or single modality measures (including volumetric) rather than utilizing complementary measures of brain function and structure that are needed to fully understand the complex brain dynamics that underlie this disorder. Project 1, which utilized several tasks and resting state, suggests that schizophrenia is characterized by inhibitory deficits that extend throughout the hemodynamic response function and across multiple tasks. Project 2 shows that although unisensory visual RTs were significantly slower for SP compared to HC, multisensory RTs (i.e., visual and auditory stimuli were presented together) did not differ by group, suggesting that the addition of the auditory stimulus helped to normalize the behavioral response in the SP group; the neurophysiological measures were consistent with this finding. Project 3 found lateralized hippocampal and PFC activation deficits in SP for nonverbal/verbal tasks, whereas a non-hippocampal elemental control task did not reveal differences between SP and HC. These results highlight the necessity of examining a hierarchy of tasks/functions across the same general set of patients/healthy controls as we did in our COBRE project. While use of rest fMRI data has become popular due to its ease of collection and use, the integration of (extended) rest fMRI data with task data is a largely understudied area. We have shown, using a hierarchy of tasks including rest, that schizophrenia patients exhibit consistent changes in connectivity from rest to tasks and in some cases show decreased connectivity at rest and increased connectivity during the most difficult tasks.

When examining linkages across modalities, in order to provide a better understanding of the structure/function networks that underlie the cognitive and social impairments experienced by SP, Project 2 results indicate that variations in MEG amplitude/timing were directly associated with alterations in WM integrity in SP versus HC. Similarly, Project 3 found lower fronto-temporal anatomical connectivity in SP, compared to HC, which was related to working-relational memory performance deficits and worse every day functioning in SP. Furthermore, our studies using multimodal functional data (e.g., MEG and fMRI), identified consistent functional components during rest, yet distinct patterns of functional network connectivity, results which emphasize the inadequacy of unimodal data collection and support the need to evaluate and integrate complex mental illness via multiple types of information. While the effect sizes for linking genome-wide individual genetic mutations with brain imaging results continue to be very small, the connection of these two modalities can give us important clues about the underlying mechanisms associated with schizophrenia. For example, multivariate association analyses can integrate multiple genetic risk loci’s effects on brain endophenotypes (Pearlson et al. 2015). Through these analyses, we were able to identify specific genetic contributions to structural abnormalities in patients with schizophrenia using a set of reference genes [Guided ICA (Chen et al. 2013) and PCA with reference (Gupta et al. 2015)]. Furthermore, we were able to demonstrate the contributions of a top schizophrenia risk gene expressing microRNA miR-137 (*MIR137*) and its targets to schizophrenia risk using pathways analyses and meta-analysis gene-set enrichment of variant associations (MAGENTA) (Wright et al. 2015, 2016) as well as gray matter concentration (Wright et al. 2016) and corpus callosum volume (Patel et al. 2015).

Ongoing advancements in computational power and algorithm development now allow us to capture some of this complexity across multiscale measures of brain function. For example, the use of dynamic (time-varying) connectivity (Calhoun et al. 2014) shows us that SPs do not always experience reduced connectivity; SP tend to spend less overall time in the more strongly connected states (Damaraju et al. 2014), which also correlates with the cortico-thalamic connectivity. Finally, we would like to emphasize the importance of data sharing and community competitions. The COBRE data competition, with over 2200 participants, demonstrated that structural and functional brain imaging is reliably predictive of schizophrenia. While this work is not yet complete, this represented one of the largest competitions demonstrating the potential for using brain imaging to inform us about diagnosis at the individual level.

## Information Sharing Statement

### Data sharing vehicle—COINS

**Fig. 9:**
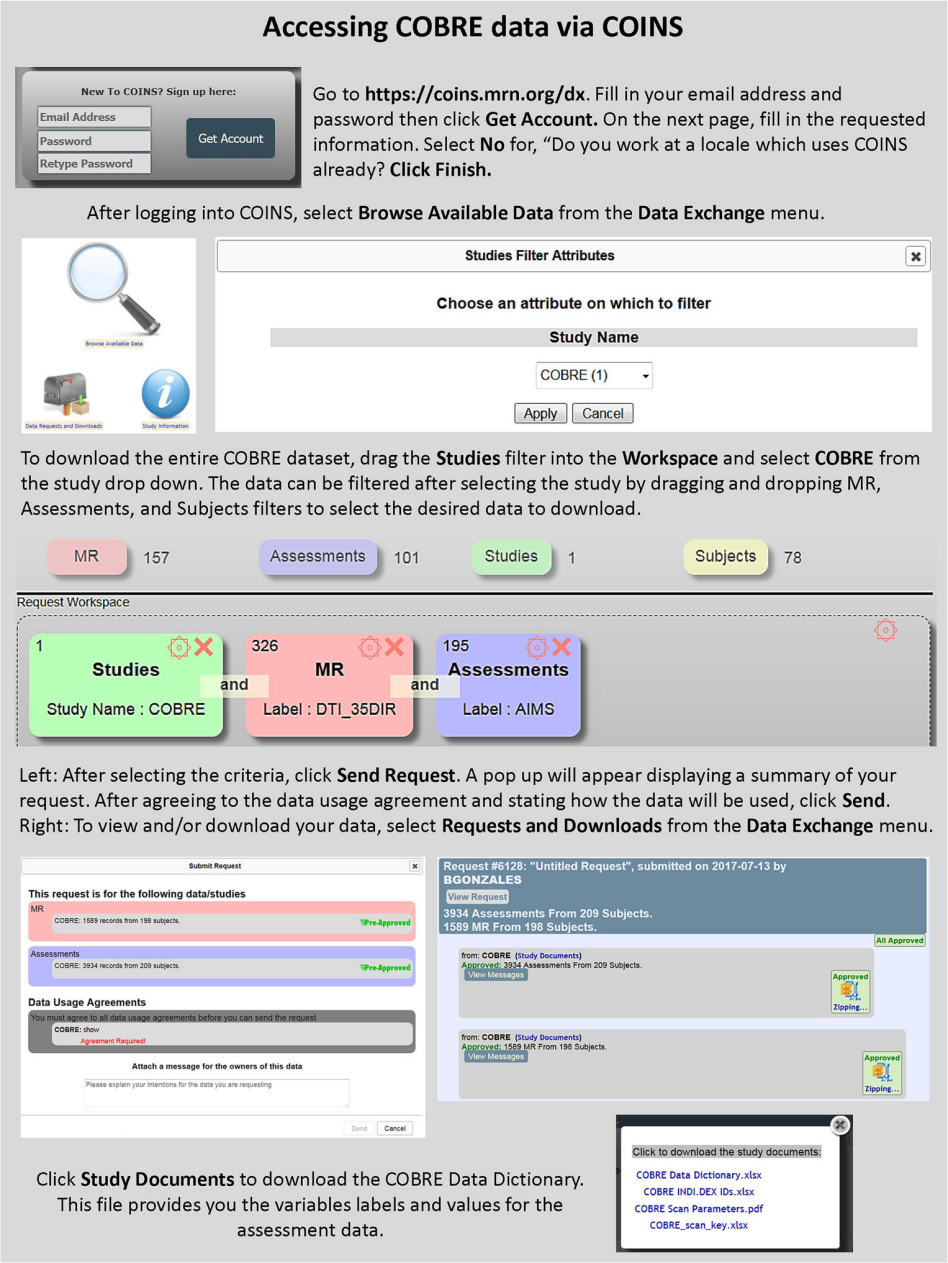
COINS webportal (coins.mrn.org/dx). Instructions for accessing data

All data are currently or will be disseminated via COINS (RRID:SCR_000805, http://coins.mrn.org; Scott et al. 2011). COINS is a mature end-to-end system for data capture and study management (King et al. 2014), archiving, and sharing (Wood et al. 2014) and serves multiple investigators and imaging centers world-wide (Bockholt et al. 2010, King et al. 2014, Scott et al. 2011, Wood et al. 2014). COINS can handle a variety of imaging modalities and analysis tools, and includes data capture and archival services that automate the transfer, organization, backup and processing of imaging data directly from the MRI or MEG scanner. For example, COINS was used to upload/share data for the CoRR consortium (over 4000 R-fMRI datasets) (Zuo et al. 2014), as well as the phenotypically rich Enhanced NKI Rockland Sample (*n* = 1000 participants) (http://fcon_1000.projects.nitrc.org/indi/enhanced/). COINS fully integrates offline and online handling of multi-site study management, radiology reading, assessments including advanced question types (King et al. 2014), DICOM image capture, and automated processing (Wood et al. 2014). Access to the shared data from COBRE (RRID:SCR_010482) and multiple other studies can be obtained by creating an account at http://coins.mrn.org/dx and searching for the desired data using the graphical query tool (Landis et al. 2016). Both assessment and imaging data are available and can be jointly downloaded (See Figure 9). For general information regarding COINS and how to access the data please contact Margaret King (mking@mrn.org). In addition, a Table listing all studies that have used COBRE data thus far is included in supplementary material (Table 2).

## Electronic supplementary material

Table 2 (DOCX 30 kb)

## References

[CR1] Abbott CC, Merideth F, Ruhl D, Yang Z, Clark VP, Calhoun VD, Hanlon FM, Mayer AR (2012). Auditory orienting and inhibition of return in schizophrenia: a functional magnetic resonance imaging study. Prog Neuro-Psychopharmacol Biol Psychiatry.

[CR2] Adler LE, Pachtman E, Franks RD, Pecevich M, Waldo MC, Freedman R (1982). Neurophysiological evidence for a defect in neuronal mechanisms involved in sensory gating in schizophrenia. Biol Psychiatry.

[CR3] Aine CJ, Sanfratello L, Adair JC, Knoefel JE, Caprihan A, Stephen JM (2011). Development and decline of memory functions in normal, pathological and healthy successful aging. Brain Topogr.

[CR4] Anderson CA, Pettersson FH, Clarke GM, Cardon LR, Morris AP, Zondervan KT (2010). Data quality control in genetic case-control association studies. Nat Protoc.

[CR5] Andreasen NC (2000). Schizophrenia: the fundamental questions. Brain Res Brain Res Rev.

[CR6] Barnes TR (1989). A rating scale for drug-induced akathisia. Br J Psychiatry.

[CR7] Bessette KL, Nave AM, Caprihan A, Stevens MC (2014). White matter abnormalities in adolescents with major depressive disorder. Brain Imaging Behav.

[CR8] Bockholt HJ, Scully M, Courtney W, Rachakonda S, Scott A, Caprihan A, Fries J, Kalyanam R, Segall JM, de la Garza R, Lane S, Calhoun VD (2010). Mining the mind research network: a novel framework for exploring large scale, heterogeneous translational neuroscience research data sources. Front Neuroinform.

[CR9] Boutros NN, Trautner P, Rosburg T, Korzyukov O, Grunwald T, Schaller C, Elger CE, Kurthen M (2005). Sensory gating in the human hippocampal and rhinal regions. Clin Neurophysiol.

[CR10] Bullmore ET, Frangou S, Murray RM (1997). The dysplastic net hypothesis: an integration of developmental and dysconnectivity theories of schizophrenia. Schizophr Res.

[CR11] Bunney WE, Bunney BG (2000). Evidence for a compromised dorsolateral prefrontal cortical parallel circuit in schizophrenia. Brain Res Brain Res Rev.

[CR12] Burns J, Job D, Bastin ME, Whalley H, Macgillivray T, Johnstone EC, Lawrie SM (2003). Structural disconnectivity in schizophrenia: a diffusion tensor magnetic resonance imaging study. Br J Psychiatry.

[CR13] Burock MA, Buckner RL, Woldorff MG, Rosen BR, Dale AM (1998). Randomized event-related experimental designs allow for extremely rapid presentation rates using functional MRI. Neuroreport.

[CR14] Butler PD, Zemon V, Schechter I, Saperstein AM, Hoptman MJ, Lim KO, Revheim N, Silipo G, Javitt DC (2005). Early-stage visual processing and cortical amplification deficits in schizophrenia. Arch Gen Psychiatry.

[CR15] Butler PD, Martinez A, Foxe JJ, Kim D, Zemon V, Silipo G, Mahoney J, Shpaner M, Jalbrzikowski M, Javitt DC (2007). Subcortical visual dysfunction in schizophrenia drives secondary cortical impairments. Brain.

[CR16] Calhoun VD, Sui J (2016). Multimodal fusion of brain imaging data: A key to finding the missing link(s) in complex illness. Biol Psychiatry: Cognitive Neuroscience and Neuroimaging.

[CR17] Calhoun VD, Miller R, Pearlson G, Adali T (2014). The chronnectome: time-varying connectivity networks as the next frontier in fMRI data discovery. Neuron.

[CR18] Caprihan A, Abbott C, Yamamoto J, Pearlson G, Perrone-Bizzozero N, Sui J, Calhoun VD (2011). Source-based morphometry analysis of group differences in fractional anisotropy in schizophrenia. Brain connectivity.

[CR19] Caprihan A, Jones T, Chen H, Lemke N, Abbott C, Qualls C, Canive J, Gasparovic C, Bustillo JR (2015). The Paradoxical Relationship between White Matter, Psychopathology and Cognition in Schizophrenia: A Diffusion Tensor and Proton Spectroscopic Imaging Study. Neuropsychopharmacology : official publication of the American College of Neuropsychopharmacology.

[CR20] Cetin MS, Christensen F, Abbott CC, Stephen JM, Mayer AR, Canive JM, Bustillo JR, Pearlson GD, Calhoun VD (2014). Thalamus and posterior temporal lobe show greater inter-network connectivity at rest and across sensory paradigms in schizophrenia. NeuroImage.

[CR21] Cetin MS, Houck JM, Rashid B, Agcaoglu O, Stephen JM, Sui J, Canive J, Mayer AR, Aine C, Bustillo JR, Calhoun VD (2016). Multimodal classification of schizophrenia patients with MEG and fMRI data using static and dynamic connectivity measures. Front Neurosci.

[CR22] Chambers JS, Perrone-Bizzozero NI (2004). Altered myelination of the hippocampal formation in subjects with schizophrenia and bipolar disorder. Neurochem Res.

[CR23] Chen J, Lin D, Hochner H (2012). Semiparametric maximum likelihood methods for analyzing genetic and environmental effects with case-control mother-child pair data. Biometrics.

[CR24] Chen J, Calhoun VD, Pearlson GD, Perrone-Bizzozero N, Sui J, Turner JA, Bustillo JR, Ehrlich S, Sponheim SR, Canive JM, Ho BC, Liu J (2013). Guided exploration of genomic risk for gray matter abnormalities in schizophrenia using parallel independent component analysis with reference. NeuroImage.

[CR25] Chua SE, McKenna PJ (1995). Schizophrenia--a brain disease? A critical review of structural and functional cerebral abnormality in the disorder. Br J Psychiatry.

[CR26] Cullum CM, Harris JG, Waldo MC, Smernoff E, Madison A, Nagamoto HT, Griffith J, Adler LE, Freedman R (1993). Neurophysiological and neuropsychological evidence for attentional dysfunction in schizophrenia. Schizophr Res.

[CR27] Damaraju E, Allen EA, Belger A, Ford JM, McEwen S, Mathalon DH, Mueller BA, Pearlson GD, Potkin SG, Preda A, Turner JA, Vaidya JG, van Erp TG, Calhoun VD (2014). Dynamic functional connectivity analysis reveals transient states of dysconnectivity in schizophrenia. Neuroimage Clin.

[CR28] Deichmann R, Gottfried JA, Hutton C, Turner R (2003). Optimized EPI for fMRI studies of the orbitofrontal cortex. NeuroImage.

[CR29] Doniger GM, Foxe JJ, Murray MM, Higgins BA, Javitt DC (2002). Impaired visual object recognition and dorsal/ventral stream interaction in schizophrenia. Arch Gen Psychiatry.

[CR30] van Erp TG, Hibar DP, Rasmussen JM, Glahn DC, Pearlson GD, Andreassen OA, Agartz I, Westlye LT, Haukvik UK, Dale AM, Melle I, Hartberg CB, Gruber O, Kraemer B, Zilles D, Donohoe G, Kelly S, McDonald C, Morris DW, Cannon DM, Corvin A, Machielsen MW, Koenders L, de Haan L, Veltman DJ, Satterthwaite TD, Wolf DH, Gur RC, Gur RE, Potkin SG, Mathalon DH, Mueller BA, Preda A, Macciardi F, Ehrlich S, Walton E, Hass J, Calhoun VD, Bockholt HJ, Sponheim SR, Shoemaker JM, van Haren NE, Pol HE, Ophoff RA, Kahn RS, Roiz-Santianez R, Crespo-Facorro B, Wang L, Alpert KI, Jonsson EG, Dimitrova R, Bois C, Whalley HC, McIntosh AM, Lawrie SM, Hashimoto R, Thompson PM, Turner JA (2016). Subcortical brain volume abnormalities in 2028 individuals with schizophrenia and 2540 healthy controls via the ENIGMA consortium. Mol Psychiatry.

[CR31] First MB, Spitzer RL, Gibbon M, Williams JBW (1995). Structured clinical interview for DSM-IV axis I disorders, non-patient edition (SCID-NP), Version 2.

[CR32] First MB, Spitzer RL, Gibbon M, Williams JBW (1995). Structured clinical interview for DSM-IV axis I disorders, patient edition (SCID-P), Version 2.

[CR33] Foxe JJ, Doniger GM, Javitt DC (2001). Early visual processing deficits in schizophrenia: impaired P1 generation revealed by high-density electrical mapping. Neuroreport.

[CR34] Foxe JJ, Murray MM, Javitt DC (2005). Filling-in in schizophrenia: a high-density electrical mapping and source-analysis investigation of illusory contour processing. Cereb Cortex.

[CR35] Freedman R, Adler LE, Gerhardt GA, Waldo M, Baker N, Rose GM, Drebing C, Nagamoto H, Bickford-Wimer P, Franks R (1987). Neurobiological studies of sensory gating in schizophrenia. Schizophr Bull.

[CR36] Freedman R, Adler LE, Myles-Worsley M, Nagamoto HT, Miller C, Kisley M, McRae K, Cawthra E, Waldo M (1996). Inhibitory gating of an evoked response to repeated auditory stimuli in schizophrenic and normal subjects. Human recordings, computer simulation, and an animal model. Arch Gen Psychiatry.

[CR37] Friston KJ (1999). Schizophrenia and the disconnection hypothesis. Acta Psychiatr Scand Suppl.

[CR38] Friston KJ (2002). Dysfunctional connectivity in schizophrenia. World Psychiatry.

[CR39] Friston KJ, Frith CD (1995). Schizophrenia: a disconnection syndrome?. Clin Neurosci.

[CR40] Gardner DM, Murphy AL, O'Donnell H, Centorrino F, Baldessarini RJ (2010). International consensus study of antipsychotic dosing. Am J Psychiatry.

[CR41] Gasparovic C, Song T, Devier D, Bockholt HJ, Caprihan A, Mullins PG, Posse S, Jung RE, Morrison LA (2006). Use of tissue water as a concentration reference for proton spectroscopic imaging. Magn Reson Med.

[CR42] Gasparovic C, Bedrick EJ, Mayer AR, Yeo RA, Chen H, Damaraju E, Calhoun VD, Jung RE (2011). Test-retest reliability and reproducibility of short-echo-time spectroscopic imaging of human brain at 3T. Magn Reson Med.

[CR43] Goldman-Rakic PS (1994). Working memory dysfunction in schizophrenia. J Neuropsychiatry Clin Neurosci.

[CR44] Goldman-Rakic PS, Selemon LD (1997). Functional and anatomical aspects of prefrontal pathology in schizophrenia. Schizophr Bull.

[CR45] Gramfort A, Luessi M, Larson E, Engemann DA, Strohmeier D, Brodbeck C, Parkkonen L, Hamalainen MS (2014). MNE software for processing MEG and EEG data. NeuroImage.

[CR46] Green MF, Nuechterlein KH (2004). The MATRICS initiative: developing a consensus cognitive battery for clinical trials. Schizophr Res.

[CR47] Green MF, Kern RS, Heaton RK (2004). Longitudinal studies of cognition and functional outcome in schizophrenia: implications for MATRICS. Schizophr Res.

[CR48] Grunwald T, Boutros NN, Pezer N, von Oertzen J, Fernandez G, Schaller C, Elger CE (2003). Neuronal substrates of sensory gating within the human brain. Biol Psychiatry.

[CR49] Gupta CN, Calhoun VD, Rachakonda S, Chen J, Patel V, Liu J, Segall J, Franke B, Zwiers MP, Arias-Vasquez A, Buitelaar J, Fisher SE, Fernandez G, van Erp TG, Potkin S, Ford J, Mathalon D, McEwen S, Lee HJ, Mueller BA, Greve DN, Andreassen O, Agartz I, Gollub RL, Sponheim SR, Ehrlich S, Wang L, Pearlson G, Glahn DC, Sprooten E, Mayer AR, Stephen J, Jung RE, Canive J, Bustillo J, Turner JA (2015). Patterns of Gray Matter Abnormalities in Schizophrenia Based on an International Mega-analysis. Schizophr Bull.

[CR50] Guy W (1976). ECDEU Assessment manual for psychopharmacology: Publication ADM 76–338.

[CR51] Hakak Y, Walker JR, Li C, Wong WH, Davis KL, Buxbaum JD, Haroutunian V, Fienberg AA (2001). Genome-wide expression analysis reveals dysregulation of myelination-related genes in chronic schizophrenia. Proc Natl Acad Sci U S A.

[CR52] Haney-Caron E, Caprihan A, Stevens MC (2014). DTI-measured white matter abnormalities in adolescents with Conduct Disorder. J Psychiatr Res.

[CR53] Hanlon FM, Weisend MP, Huang M, Lee RR, Moses SN, Paulson KM, Thoma RJ, Miller GA, Canive JM (2003). A non-invasive method for observing hippocampal function. Neuroreport.

[CR54] Hanlon FM, Miller GA, Thoma RJ, Irwin J, Jones A, Moses SN, Huang M, Weisend MP, Paulson KM, Edgar JC, Adler LE, Canive JM (2005). Distinct M50 and M100 auditory gating deficits in schizophrenia. Psychophysiology.

[CR55] Hanlon FM, Weisend MP, Yeo RA, Huang M, Lee RR, Thoma RJ, Moses SN, Paulson KM, Miller GA, Canive JM (2005). A specific test of hippocampal deficit in schizophrenia. Behav Neurosci.

[CR56] Hanlon FM, Weisend MP, Hamilton DA, Jones AP, Thoma RJ, Huang M, Martin K, Yeo RA, Miller GA, Canive JM (2006). Impairment on the hippocampal-dependent virtual Morris water task in schizophrenia. Schizophr Res.

[CR57] Hanlon FM, Houck JM, Pyeatt CJ, Lundy SL, Euler MJ, Weisend MP, Thoma RJ, Bustillo JR, Miller GA, Tesche CD (2011). Bilateral hippocampal dysfunction in schizophrenia. NeuroImage.

[CR58] Hanlon FM, Houck JM, Klimaj SD, Caprihan A, Mayer AR, Weisend MP, Bustillo JR, Hamilton DA, Tesche CD (2012). Frontotemporal anatomical connectivity and working-relational memory performance predict everyday functioning in schizophrenia. Psychophysiology.

[CR59] Hanlon FM, Shaff NA, Dodd AB, Ling JM, Bustillo JR, Abbott CC, Stromberg SF, Abrams S, Lin DS, Mayer AR (2016). Hemodynamic response function abnormalities in schizophrenia during a multisensory detection task. Hum Brain Mapp.

[CR60] Hof PR, Haroutunian V, Copland C, Davis KL, Buxbaum JD (2002). Molecular and cellular evidence for an oligodendrocyte abnormality in schizophrenia. Neurochem Res.

[CR61] Hof PR, Haroutunian V, Friedrich VL, Byne W, Buitron C, Perl DP, Davis KL (2003). Loss and altered spatial distribution of oligodendrocytes in the superior frontal gyrus in schizophrenia. Biol Psychiatry.

[CR62] Honey GD, Fletcher PC (2006). Investigating principles of human brain function underlying working memory: what insights from schizophrenia?. Neuroscience.

[CR63] Houck, J. M., Cetin, M. S., Mayer, A. R., Bustillo, J. R., Stephen, J., Aine, C. J., Canive, J., Perrone-Bizzozero, N., Thoma, R. J., Brookes, M. J., & Calhoun, V. D. (2017). Magnetoencephalographic and functional MRI connectomics in schizophrenia via intra- and inter-network connectivity. *NeuroImage, 145*(Pt A), 96–106. 10.1016/j.neuroimage.2016.10.011 PMC5179295 27725313

[CR64] Huang MX, Edgar JC, Thoma RJ, Hanlon FM, Moses SN, Lee RR, Paulson KM, Weisend MP, Irwin JG, Bustillo JR, Adler LE, Miller GA, Canive JM (2003). Predicting EEG responses using MEG sources in superior temporal gyrus reveals source asynchrony in patients with schizophrenia. Clin Neurophysiol.

[CR65] Johnson JD (2006). The conversational brain: fronto-hippocampal interaction and disconnection. Med Hypotheses.

[CR66] Johnstone EC, Frith CD, Crow TJ, Husband J, Kreel L (1976). Cerebral Ventricular Size and Cognitive Impairment in Chronic Schizophrenia. Lancet.

[CR67] Jones DK, Horsfield MA, Simmons A (1999). Optimal strategies for measuring diffusion in anisotropic systems by magnetic resonance imaging. Magn Reson Med.

[CR68] Jung RE, Haier RJ (2007). The Parieto-Frontal Integration Theory (P-FIT) of intelligence: Converging neuroimaging evidence. Behav Brain Sci.

[CR69] Kalyanam D, Boutte C, Gasparovic C, Hutchinson KE, Calhoun VD (2013). Group independent component analysis of MR spectra. Brain Imaging and Behavior.

[CR70] Kalyanam, D., Boutte, C., Hutchinson, K. E., & Calhoun, V. D. (2015). Application of ICA to realistically simulated 1H-MRS data. *Brain and Behavior, 5*(7), e00345. 10.1002/brb3.345 PMC4511286 26221570

[CR71] Kay SR, Fiszbein A, Opler LA (1987). The positive and negative syndrome scale (PANSS) for schizophrenia. Schizophr Bull.

[CR72] Kern RS, Green MF, Nuechterlein KH, Deng BH (2004). NIMH-MATRICS survey on assessment of neurocognition in schizophrenia. Schizophr Res.

[CR73] Kim D, Zemon V, Saperstein A, Butler PD, Javitt DC (2005). Dysfunction of early-stage visual processing in schizophrenia: harmonic analysis. Schizophr Res.

[CR74] Kim D, Wylie G, Pasternak R, Butler PD, Javitt DC (2006). Magnocellular contributions to impaired motion processing in schizophrenia. Schizophr Res.

[CR75] King MD, Wood D, Miller B, Kelly R, Landis D, Courtney W, Wang R, Turner JA, Calhoun VD (2014). Automated collection of imaging and phenotypic data to centralized and distributed data repositories. Front Neuroinform.

[CR76] Korzyukov O, Pflieger ME, Wagner M, Bowyer SM, Rosburg T, Sundaresan K, Elger CE, Boutros NN (2007). Generators of the intracranial P50 response in auditory sensory gating. NeuroImage.

[CR77] Kraepelin E (1896). Lehrbuch der Psychiatrie.

[CR78] Kubicki M, Westin CF, Maier SE, Frumin M, Nestor PG, Salisbury DF, Kikinis R, Jolesz FA, McCarley RW, Shenton ME (2002). Uncinate fasciculus findings in schizophrenia: a magnetic resonance diffusion tensor imaging study. Am J Psychiatry.

[CR79] Kuhn S, Gallinat J (2013). Resting-state brain activity in schizophrenia and major depression: a quantitative meta-analysis. Schizophr Bull.

[CR80] Landis D, Courtney W, Dieringer C, Kelly R, King M, Miller B, Wang R, Wood D, Turner JA, Calhoun VD (2016). COINS Data Exchange: An open platform for compiling, curating, and disseminating neuroimaging data. NeuroImage.

[CR81] Lijffijt M, Moeller FG, Boutros NN, Steinberg JL, Meier SL, Lane SD, Swann AC (2009). Diminished P50, N100 and P200 auditory sensory gating in bipolar I disorder. Psychiatry Res.

[CR82] Liu X, Qin W, He G, Yang Y, Chen Q, Zhou J, Li D, Gu N, Xu Y, Feng G, Sang H, Hao X, Zhang K, Wang S, He L (2005). A family-based association study of the MOG gene with schizophrenia in the Chinese population. Schizophr Res.

[CR83] Mayer AR, Ruhl D, Merideth F, Ling J, Hanlon FM, Bustillo J, Canive J (2013). Functional imaging of the hemodynamic sensory gating response in schizophrenia. Hum Brain Mapp.

[CR84] Mayer AR, Hanlon FM, Teshiba TM, Klimaj SD, Ling JM, Dodd AB, Calhoun VD, Bustillo JR, Toulouse T (2015). An fMRI study of multimodal selective attention in schizophrenia. Br J Psychiatry.

[CR85] Mayer AR, Hanlon FM, Dodd AB, Yeo RA, Haaland KY, Ling JM, Ryman SG (2016). Proactive response inhibition abnormalities in the sensorimotor cortex of patients with schizophrenia. J Psychiatry Neurosci.

[CR86] Monnig MA, Caprihan A, Yeo RA, Gasparovic C, Ruhl DA, Lysne P, Bogenschutz MP, Hutchison KE, Thoma RJ (2013). Diffusion tensor imaging of white matter networks in individuals with current and remitted alcohol use disorders and comorbid conditions. Psychology of addictive behaviors : journal of the Society of Psychologists in Addictive Behaviors.

[CR87] Mullins PG, Chen H, Xu J, Caprihan A, Gasparovic C (2008). Comparative reliability of proton spectroscopy techniques designed to improve detection of J-coupled metabolites. Magn Reson Med.

[CR88] Novak G, Kim D, Seeman P, Tallerico T (2002). Schizophrenia and Nogo: elevated mRNA in cortex, and high prevalence of a homozygous CAA insert. Brain Res Mol Brain Res.

[CR89] O'Donnell BF, Swearer JM, Smith LT, Nestor PG, Shenton ME, McCarley RW (1996). Selective deficits in visual perception and recognition in schizophrenia. Am J Psychiatry.

[CR90] Orlovskaya D, Denisov D, Uranova NA (1997). The ultrastructural pathology of myelinated fibers and oligodendroglial cells in autopsied caudate nucleus of schizophrenics. Schizophr Res.

[CR91] Orlovskaya D, Vikhreva O, Zimina IS, Denisov DV, Uranova NA (1999). Ultrastructural dystrophic changes of oligodendroglial cells in autopsied prefrontal cortex and striatum in schizophrenia: a morphometric study. Schizophr Res.

[CR92] Park HJ, Westin CF, Kubicki M, Maier SE, Niznikiewicz M, Baer A, Frumin M, Kikinis R, Jolesz FA, McCarley RW, Shenton ME (2004). White matter hemisphere asymmetries in healthy subjects and in schizophrenia: a diffusion tensor MRI study. NeuroImage.

[CR93] Pascual-Marqui RD (2002). Standardized low-resolution brain electromagnetic tomography (sLORETA): technical details. Methods Find Exp Clin Pharmacol.

[CR94] Patel VS, Kelly S, Wright C, Gupta CN, Arias-Vasquez A, Perrone-Bizzozero N, Ehrlich S, Wang L, Bustillo JR, Morris D, Corvin A, Cannon DM, McDonald C, Donohoe G, Calhoun VD, Turner JA (2015). MIR137HG risk variant rs1625579 genotype is related to corpus callosum volume in schizophrenia. Neurosci Lett.

[CR95] Patterson TL, Goldman S, McKibbin CL, Hughs T, Jeste DV (2001). UCSD Performance-Based Skills Assessment: development of a new measure of everyday functioning for severely mentally ill adults. Schizophr Bull.

[CR96] Patterson JV, Hetrick WP, Boutros NN, Jin Y, Sandman C, Stern H, Potkin S, Bunney WE (2008). P50 sensory gating ratios in schizophrenics and controls: a review and data analysis. Psychiatry Res.

[CR97] Pearlson GD, Liu J, Calhoun VD (2015). An introductory review of parallel independent component analysis (p-ICA) and a guide to applying p-ICA to genetic data and imaging phenotypes to identify disease-associated biological pathways and systems in common complex disorders. Front Genet.

[CR98] Peirce TR, Bray NJ, Williams NM, Norton N, Moskvina V, Preece A, Haroutunian V, Buxbaum JD, Owen MJ, O'Donovan MC (2006). Convergent evidence for 2′,3′-cyclic nucleotide 3′-phosphodiesterase as a possible susceptibility gene for schizophrenia. Arch Gen Psychiatry.

[CR99] Purcell S, Daly MJ, Sham PC (2007). WHAP: haplotype-based association analysis. Bioinformatics.

[CR100] Qin W, Gao J, Xing Q, Yang J, Qian X, Li X, Guo Z, Chen H, Wang L, Huang X, Gu N, Feng G, He L (2005). A family-based association study of PLP1 and schizophrenia. Neurosci Lett.

[CR101] Ripke S, O'Dushlaine C, Chambert K, Moran JL, Kahler AK, Akterin S, Bergen SE, Collins AL, Crowley JJ, Fromer M, Kim Y, Lee SH, Magnusson PK, Sanchez N, Stahl EA, Williams S, Wray NR, Xia K, Bettella F, Borglum AD, Bulik-Sullivan BK, Cormican P, Craddock N, de Leeuw C, Durmishi N, Gill M, Golimbet V, Hamshere ML, Holmans P, Hougaard DM, Kendler KS, Lin K, Morris DW, Mors O, Mortensen PB, Neale BM, O'Neill FA, Owen MJ, Milovancevic MP, Posthuma D, Powell J, Richards AL, Riley BP, Ruderfer D, Rujescu D, Sigurdsson E, Silagadze T, Smit AB, Stefansson H, Steinberg S, Suvisaari J, Tosato S, Verhage M, Walters JT, Levinson DF, Gejman PV, Kendler KS, Laurent C, Mowry BJ, O'Donovan MC, Owen MJ, Pulver AE, Riley BP, Schwab SG, Wildenauer DB, Dudbridge F, Holmans P, Shi J, Albus M, Alexander M, Campion D, Cohen D, Dikeos D, Duan J, Eichhammer P, Godard S, Hansen M, Lerer FB, Liang KY, Maier W, Mallet J, Nertney DA, Nestadt G, Norton N, O'Neill FA, Papadimitriou GN, Ribble R, Sanders AR, Silverman JM, Walsh D, Williams NM, Wormley B, Arranz MJ, Bakker S, Bender S, Bramon E, Collier D, Crespo-Facorro B, Hall J, Iyegbe C, Jablensky A, Kahn RS, Kalaydjieva L, Lawrie S, Lewis CM, Lin K, Linszen DH, Mata I, McIntosh A, Murray RM, Ophoff RA, Powell J, Rujescu D, Van Os J, Walshe M, Weisbrod M, Wiersma D, Donnelly P, Barroso I, Blackwell JM, Bramon E, Brown MA, Casas JP, Corvin AP, Deloukas P, Duncanson A, Jankowski J, Markus HS, Mathew CG, Palmer CN, Plomin R, Rautanen A, Sawcer SJ, Trembath RC, Viswanathan AC, Wood NW, Spencer CC, Band G, Bellenguez C, Freeman C, Hellenthal G, Giannoulatou E, Pirinen M, Pearson RD, Strange A, Su Z, Vukcevic D, Donnelly P, Langford C, Hunt SE, Edkins S, Gwilliam R, Blackburn H, Bumpstead SJ, Dronov S, Gillman M, Gray E, Hammond N, Jayakumar A, McCann OT, Liddle J, Potter SC, Ravindrarajah R, Ricketts M, Tashakkori-Ghanbaria A, Waller MJ, Weston P, Widaa S, Whittaker P, Barroso I, Deloukas P, Mathew CG, Blackwell JM, Brown MA, Corvin AP, McCarthy MI, Spencer CC, Bramon E, Corvin AP, O'Donovan MC, Stefansson K, Scolnick E, Purcell S, McCarroll SA, Sklar P, Hultman CM, Sullivan PF, Multicenter Genetic Studies of Schizophrenia, C, Psychosis Endophenotypes International, C, Wellcome Trust Case Control, C (2013). Genome-wide association analysis identifies 13 new risk loci for schizophrenia. Nat Genet.

[CR102] Schechter I, Butler PD, Zemon VM, Revheim N, Saperstein AM, Jalbrzikowski M, Pasternak R, Silipo G, Javitt DC (2005). Impairments in generation of early-stage transient visual evoked potentials to magno- and parvocellular-selective stimuli in schizophrenia. Clin Neurophysiol.

[CR103] Schechter I, Butler PD, Jalbrzikowski M, Pasternak R, Saperstein AM, Javitt DC (2006). A new dimension of sensory dysfunction: stereopsis deficits in schizophrenia. Biol Psychiatry.

[CR104] Schooler NR, Kane JM (1982). Research diagnoses for tardive dyskinesia. Arch Gen Psychiatry.

[CR105] Schroeder CE, Foxe JJ (2002). The timing and laminar profile of converging inputs to multisensory areas of the macaque neocortex. Brain Res Cogn Brain Res.

[CR106] Schroeder CE, Lindsley RW, Specht C, Marcovici A, Smiley JF, Javitt DC (2001). Somatosensory input to auditory association cortex in the macaque monkey. J Neurophysiol.

[CR107] Scott A, Courtney W, Wood D, de la Garza R, Lane S, King M, Wang R, Roberts J, Turner JA, Calhoun VD (2011). COINS: An Innovative Informatics and Neuroimaging Tool Suite Built for Large Heterogeneous Datasets. Front Neuroinform.

[CR108] Segall JM, Allen EA, Jung RE, Erhardt EB, Arja SK, Kiehl K, Calhoun VD (2012). Correspondence between structure and function in the human brain at rest. Front Neuroinform.

[CR109] Silva R, Castro E, Gupta N, Cetin M, Arbabshirani M, Potluru V, Plis SM, Calhoun VD, "The Tenth Annual MLSP Competition: Schizophrenia Classification Challenge," in *IEEE International Workshop on Machine Learning for Signal Processing*. IEEE Reims, France, Sept. 21-24, 2014.

[CR110] Simpson GM, Angus JW (1970). A rating scale for extrapyramidal side effects. Acta Psychiatr Scand Suppl.

[CR111] Smith SM, Jenkinson M, Johansen-Berg H, Rueckert D, Nichols TE, MacKay CE, Watkins KE, Ciccarelli O, Cader MZ, Mathews PM, Behrens TEJ (2006). Tract-based spatial statistics: Voxelwise analysis of multi-subject diffusion data. NeuroImage.

[CR112] Stanford TR, Stein BE (2007). Superadditivity in multisensory integration: putting the computation in context. Neuroreport.

[CR113] Stein BE, Meredith MA, Wallace MT (1993). The visually responsive neuron and beyond: multisensory integration in cat and monkey. Prog Brain Res.

[CR114] Stephan KE, Baldeweg T, Friston KJ (2006). Synaptic plasticity and dysconnection in schizophrenia. Biol Psychiatry.

[CR115] Stephen JM, Coffman BA, Jung RE, Bustillo JR, Aine CJ, Calhoun VD (2013). Using joint ICA to link function and structure using MEG and DTI in schizophrenia. NeuroImage.

[CR116] Stone DB, Urrea LJ, Aine CJ, Bustillo JR, Clark VP, Stephen JM (2011). Unisensory processing and multisensory integration in schizophrenia: a high-density electrical mapping study. Neuropsychologia.

[CR117] Stone DB, Coffman BA, Bustillo JR, Aine CJ, Stephen JM (2014). Multisensory stimuli elicit altered oscillatory brain responses at gamma frequencies in patients with schizophrenia. Front Hum Neurosci.

[CR118] Sui J, Pearlson GD, Du Y, Yu Q, Jones TR, Chen J, Jiang T, Bustillo J, Calhoun VD (2015). In search of multimodal neuroimaging biomarkers of cognitive deficits in schizophrenia. Biol Psychiatry.

[CR119] Talairach J, Tournoux P (1988). Co-lanar stereotaxic atlas of the human brain.

[CR120] Tan EC, Chong SA, Wang H, Chew-Ping Lim E, Teo YY (2005). Gender-specific association of insertion/deletion polymorphisms in the nogo gene and chronic schizophrenia. Brain Res Mol Brain Res.

[CR121] Thoma RJ, Hanlon FM, Moses SN, Edgar JC, Huang M, Weisend MP, Irwin J, Sherwood A, Paulson K, Bustillo J, Adler LE, Miller GA, Canive JM (2003). Lateralization of auditory sensory gating and neuropsychological dysfunction in schizophrenia. Am J Psychiatry.

[CR122] Thoma RJ, Hanlon FM, Moses SN, Ricker D, Huang M, Edgar C, Irwin J, Torres F, Weisend MP, Adler LE, Miller GA, Canive JM (2005). M50 sensory gating predicts negative symptoms in schizophrenia. Schizophr Res.

[CR123] Uranova N, Orlovskaya D, Vikhreva O, Zimina I, Kolomeets N, Vostrikov V, Rachmanova V (2001). Electron microscopy of oligodendroglia in severe mental illness. Brain Res Bull.

[CR124] Uusitalo MA, Ilmoniemi RJ (1997). Signal-space projection method for separating MEG or EEG into components. Med Biol Eng Comput.

[CR125] Vakhtin AA, Ryman SG, Flores RA, Jung RE (2014). Functional brain networks contributing to the Parieto-Frontal Integration Theory of Intelligence. NeuroImage.

[CR126] Wagner M, Fuchs M, Kastner J (2004). Evaluation of sLORETA in the presence of noise and multiple sources. Brain Topogr.

[CR127] Wakana S, Jiang H, Nagae-Poetscher LM, van Zijl PC, Mori S (2004). Fiber tract-based atlas of human white matter anatomy. Radiology.

[CR128] Wan C, Yang Y, Feng G, Gu N, Liu H, Zhu S, He L, Wang L (2005). Polymorphisms of myelin-associated glycoprotein gene are associated with schizophrenia in the Chinese Han population. Neurosci Lett.

[CR129] Williams LE, Light GA, Braff DL, Ramachandran VS (2010). Reduced multisensory integration in patients with schizophrenia on a target detection task. Neuropsychologia.

[CR130] Wolf RC, Vasic N, Sambataro F, Hose A, Frasch K, Schmid M, Walter H (2009). Temporally anticorrelated brain networks during working memory performance reveal aberrant prefrontal and hippocampal connectivity in patients with schizophrenia. Prog Neuro-Psychopharmacol Biol Psychiatry.

[CR131] Wood D, King M, Landis D, Courtney W, Wang R, Kelly R, Turner JA, Calhoun VD (2014). Harnessing modern web application technology to create intuitive and efficient data visualization and sharing tools. Front Neuroinform.

[CR132] Wright C, Calhoun VD, Ehrlich S, Wang L, Turner JA, Bizzozero NI (2015). Meta gene set enrichment analyses link miR-137-regulated pathways with schizophrenia risk. Front Genet.

[CR133] Wright C, Gupta CN, Chen J, Patel V, Calhoun VD, Ehrlich S, Wang L, Bustillo JR, Perrone-Bizzozero NI, Turner JA (2016). Polymorphisms in MIR137HG and microRNA-137-regulated genes influence gray matter structure in schizophrenia. Transl Psychiatry.

[CR134] Wu L, Calhoun VD, Jung RE, Caprihan A (2015). Connectivity-based whole brain dual parcellation by group ICA reveals tract structures and decreased connectivity in schizophrenia. Hum Brain Mapp.

[CR135] Zuo XN, Anderson JS, Bellec P, Birn RM, Biswal BB, Blautzik J, Breitner JC, Buckner RL, Calhoun VD, Castellanos FX, Chen A, Chen B, Chen J, Chen X, Colcombe SJ, Courtney W, Craddock RC, Di Martino A, Dong HM, Fu X, Gong Q, Gorgolewski KJ, Han Y, He Y, He Y, Ho E, Holmes A, Hou XH, Huckins J, Jiang T, Jiang Y, Kelley W, Kelly C, King M, LaConte SM, Lainhart JE, Lei X, Li HJ, Li K, Li K, Lin Q, Liu D, Liu J, Liu X, Liu Y, Lu G, Lu J, Luna B, Luo J, Lurie D, Mao Y, Margulies DS, Mayer AR, Meindl T, Meyerand ME, Nan W, Nielsen JA, O'Connor D, Paulsen D, Prabhakaran V, Qi Z, Qiu J, Shao C, Shehzad Z, Tang W, Villringer A, Wang H, Wang K, Wei D, Wei GX, Weng XC, Wu X, Xu T, Yang N, Yang Z, Zang YF, Zhang L, Zhang Q, Zhang Z, Zhang Z, Zhao K, Zhen Z, Zhou Y, Zhu XT, Milham MP (2014). An open science resource for establishing reliability and reproducibility in functional connectomics. Sci Data.

