# Supplementary material for: Multimodal Neuroimaging in Schizophrenia: Description and Dissemination
Source: Neuroinformatics. 2017 Aug 15;15(4):343–64. doi: 10.1007/s12021-017-9338-9 (PMC5671541; doi:10.1007/s12021-017-9338-9)
Supplement: Supplementary file 1 — (DOCX 30 kb) [file 12021_2017_9338_MOESM1_ESM.docx]

TABLE 2

| Abbott CC, Merideth F, Ruhl D, Yan, Z, Clark VP, Calhoun VD, Hanlon FM, Mayer AR. Auditory orienting and inhibition of return in schizophrenia: a functional magnetic resonance imaging study. Prog Neuropsychopharmacol Biol Psychiatry. 37(1):161-8, 2012. |
| --- |
| Adams HH, Hibar DP, Chouraki V, Stein JL, Nyquist PA, Rentería ME, Trompet S, Arias-Vasquez A, Seshadri S, Desrivières S, Beecham AH, Jahanshad N, Wittfeld K, Van der Lee SJ, Abramovic L, Alhusaini S, Amin N, Andersson M, Arfanakis K, Aribisala BS, Armstrong NJ, Athanasiu L, Axelsson T, Beiser A, Bernard M, Bis JC, Blanken LM, Blanton SH, Bohlken, MM, Boks MP, Bralten J, Brickman AM, Carmichael O, Chakravarty MM, Chauhan G, Chen Q, Ching CR, Cuellar-Partida, G, Braber AD, Doan NT, Ehrlich S, Filippi I, Ge T, Giddaluru S, Goldman AL, Gottesman RF, Greven CU, Grimm O, Griswold ME, Guadalupe T, Hass J, Haukvik UK, Hilal S, Hofer E, Hoehn D, Holmes AJ, et al. [Novel genetic loci underlying human intracranial volume identified through genome-wide association.](https://www.ncbi.nlm.nih.gov/pubmed/27694991) Nat Neurosci. Oct 3 2016. doi: 10.1038/nn.4398. |
| Aine CJ, Sanfratello L, Ranken D, Best E, MacArthur JA, Wallace T, Gilliam K, Donahue CH, Montaño R, Bryant JE, Scott A, Stephen JM. MEG-SIM: a web portal for testing MEG analysis methods using realistic simulated and empirical data. Neuroinformatics. 10(2):141-58, 2012. |
| Anderson A, Cohen MS. Decreased small-world functional network connectivity and clustering across resting state networks in schizophrenia: an fMRI classification tutorial. Front Hum Neurosci. Sep2;7:520. 2013, PMID 24032010. |
| Bellec P, Benhajali Y, Carbonell F, Dansereau C, Albouy G, Pelland M, Craddock C, Collignon O, Doyon J, Stip E, Orban P. Impact of the resolution of brain parcels on connectome-wide association studies in fMRI. Neuroimage. 123:212-28, 2015. |
| Bockholt HJ, Scully, M, Courtney, W, Rachakonda, S, Scott, A, Caprihan, A, Fries, J, Kalyanam, R, Segall, JM, de la Garza, R, Lane, S, Calhoun, VD. Mining the mind research network: a novel framework for exploring large scale, heterogeneous translational neuroscience research data sources. Front Neuroinform 3: 36, 2010. |
| Caprihan A, Abbott C, Yamamoto J, Pearlson G, Perrone-Bizzozero N, Sui J, Calhoun VD. Source-based morphometry analysis of group differences in fractional anisotropy in schizophrenia. Brain connectivity. 1:133-145, 2011. |
| Caprihan A, Jones T, Chen H, Lemke N, Abbott C, Qualls C, Canive J, Gasparovic C, Bustillo JR. The Paradoxical Relationship between White Matter, Psychopathology and Cognition in Schizophrenia: A Diffusion Tensor and Proton Spectroscopic Imaging Study. Neuropsychopharmacology. 40:2248-2257, 2015. |
| Calhoun VD, Adalı T. Multi-subject Independent Component Analysis of fMRI: A Decade of Intrinsic Networks, Default Mode, and Neurodiagnostic Discovery, IEEE Reviews in Biomedical Engineering. 5:60-73, 2012, PMC23231989. |
| Calhoun VD, Miller R, Pearlson, G, Adali, T. The chronnectome: time-varying connectivity networks as the next frontier in fMRI data discovery, Neuron. 84:262-274, 2014, PMC4372723. |
| Calhoun VD, Sui J. Multimodal fusion of brain imaging data: A key to finding the missing link(s) in complex mental illness," Biological Psychiatry: Cognitive Neuroscience and Neuroimaging. 1(3):230-244, 2016. |
| Çetin MS, Christensen F, Abbott CC, Stephen JM, Mayer AR, Cañive JM, Bustillo JR, Pearlson GD, Calhoun VD. Thalamus and posterior temporal lobe show greater inter-network connectivity at rest and across sensory paradigms in schizophrenia. Neuroimage, 97:117-126, 2014. |
| Çetin MS, Houck JM, Rashid B, Agcaoglu O, Stephen J, Sui J, Cañive J, Aine C, Bustillo J, Calhoun VD. Multimodal classification of schizophrenia patients with MEG and fMRI data using static and dynamic connectivity measures. Frontiers Neurosci. Oct 19;10:466, 2016. |
| Chen J, Calhoun VD, Pearlson GD, Ehrlich S, Turner JA, Ho BC, Wassink TH, Michael AM, Liu J. [Multifaceted genomic risk for brain function in schizophrenia.](https://www.ncbi.nlm.nih.gov/pubmed/22440650) Neuroimage. 61(4):866-75, 2012. |
| Chen J, Calhoun VD, Pearlson GD, Perrone-Bizzozero N, Sui J, Turner JA, Bustillo JR, Ehrlich S, Sponheim SR, Cañive JM, Ho BC, Liu J. [Guided exploration of genomic risk for gray matter abnormalities in schizophrenia using parallel independent component analysis with reference.](http://www.ncbi.nlm.nih.gov/pubmed/23727316) Neuroimage. 83:384-96, 2013. |
| Chen J, Calhoun VD, Pearlson GD, Perrone-Bizzozero NI, Turner JA, Ehrlich S, Ho BC, Liu J. [Independent component analysis of SNPs reflects polygenic risk scores for schizophrenia.](https://www.ncbi.nlm.nih.gov/pubmed/27637363) Schizophr Res. Sep 13, 2016. pii: S0920-9964(16)30393-0. |
| Chen J, Calhoun VD, Perrone-Bizzozero NI, Pearlson GD, Sui J, Du Y, Liu J.[A pilot study on commonality and specificity of copy number variants in schizophrenia and bipolar disorder.](https://www.ncbi.nlm.nih.gov/pubmed/27244233) Transl Psychiatry. May 31;6(5):e824, 2016. |
| Chen J, Calhoun VD, Ulloa AE, Liu J. [Parallel ICA with multiple references: a semi-blind multivariate approach.](https://www.ncbi.nlm.nih.gov/pubmed/25571523) Conf Proc IEEE Eng Med Biol Soc. 2014;2014:6659-62. |
| Chen J, Liu J, Calhoun VD, Arias-Vasquez A, Zwiers MP, Gupta CN, Franke B, Turner JA. Exploration of scanning effects in multi-site structural MRI studies. J Neurosci Methods. 230:37-50, 2014. |
| Demirci O, Stevens MC, Andreasen NC, Michael A, Liu J, White T, Pearlson GD, Clark VP, Calhoun VD. [Investigation of relationships between fMRI brain networks in the spectral domain using ICA and Granger causality reveals distinct differences between schizophrenia patients and healthy controls.](https://www.ncbi.nlm.nih.gov/pubmed/19245841) Neuroimage. 46(2):419-31, 2009. |
| Du Y, Liu J, Sui J, He H, Pearlson GD, Calhoun VD. [Exploring difference and overlap between schizophrenia, schizoaffective and bipolar disorders using resting-state brain functional networks.](https://www.ncbi.nlm.nih.gov/pubmed/25570258) Conf Proc IEEE Eng Med Biol Soc. 2014;2014:1517-20. |
| Du Y, Pearlson GD, Liu J, Sui J, Yu Q, He H, Castro E, Calhoun VD. [A group ICA based framework for evaluating resting fMRI markers when disease categories are unclear: application to schizophrenia, bipolar, and schizoaffective disorders.](https://www.ncbi.nlm.nih.gov/pubmed/26216278) Neuroimage. 122:272-80, 2015. |
| Erhardt E, Allen E, Wei Y, Eichele T Calhoun VD. SimTB, a simulation toolbox for fMRI data under a model of spatiotemporal separability," Neuroimage. 59:4160-4167, 2012, PMC3690331. |
| Gasparovic C, Song T, Devier D, Bockholt HJ, Caprihan A, Mullins PG, et al. Use of tissue water as a concentration reference for proton spectroscopic imaging. Magn Reson Med. 55(6):1219-26, 2006, PMID: 16688703. |
| Gasparovic C, Bedrick EJ, Mayer AR, Yeo RA, Chen H, Damaraju E, et al. Test-retest reliability and reproducibility of short-echo-time spectroscopic imaging of human brain at 3T. Magn Reson Med. 66(2):324-32, 2011, PMCID: 3130105. |
| Gupta CN, Calhoun VD, Rachakonda S, Chen J, Patel V, Liu J, Segall J, Franke B, Zwiers MP, Arias-Vasquez A, Buitelaar J, Fisher SE, Fernandez G, van Erp TG, Potkin S, Ford J, Mathalon D, McEwen S, Lee HJ, Mueller BA, Greve DN, Andreassen O, Agartz I, Gollub RL, Sponheim SR, Ehrlich S, Wang L, Pearlson G, Glahn DC, Sprooten E, Mayer AR, Stephen J, Jung RE, Canive J, Bustillo J, Turner JA. Patterns of Gray Matter Abnormalities in Schizophrenia Based on an International Mega-analysis. Schizophr Bull. 41(5):1133-42, 2015. |
| Gupta CN, Chen J, Liu J, Damaraju E, Wright C, Perrone-Bizzozero NI, Pearlson G, Luo L, Michael AM, Turner JA, Calhoun VD. [Genetic markers of white matter integrity in schizophrenia revealed by parallel ICA.](https://www.ncbi.nlm.nih.gov/pubmed/25784871) Front Hum Neurosci. Mar 3, 2015;9:100. |
| Haney-Caron E, Caprihan A, Stevens MC. DTI-measured white matter abnormalities in adolescents with Conduct Disorder. J Psychiatr Res. 48:111-120, 2014. |
| Hanlon FM, Houck JM, Pyeatt CJ, Lundy SL, Euler MJ, Weisend MP, Thoma RJ, Bustillo JR, Miller GA, Tesche CD. Bilateral hippocampal dysfunction in schizophrenia. Neuroimage. 58(4):1158-68, 2011. |
| Hanlon FM, Houck JM, Klimaj SD, Caprihan A, Mayer AR, Weisend MP, Bustillo JR, Hamilton DA, Tesche CD. Frontotemporal anatomical connectivity and working-relational memory performance predict everyday functioning in schizophrenia. Psychophysiology. 49(10):1340-52, 2012. |
| Hanlon FM, Shaff NA, Dodd AB, Ling JM, Bustillo JR, Abbott CC, Stromberg SF, Abrams S, Lin DS, Mayer AR. Hemodynamic response function abnormalities in schizophrenia during a multisensory detection task. Hum Brain Mapp. 37(2):745-55, 2016. |
| Hass J, Walton E, Kirsten H, Liu J, Priebe L, Wolf C, Karbalai N, Gollub R, White T, Roessner V, Müller KU, Paus T, Smolka MN, Schumann G, IMAGEN Consortium, Scholz M, Cichon S, Calhoun V, Ehrlich S. [A Genome-Wide Association Study Suggests Novel Loci Associated with a Schizophrenia-Related Brain-Based Phenotype.](https://www.ncbi.nlm.nih.gov/pubmed/23805179) PLoS One. Jun 21, 2013;8(6):e64872. |
| Hass J, Walton E, Wright C, Beyer A, Scholz M, Turner J, Liu J, Smolka MN, Roessner V, Sponheim SR, Gollub RL, Calhoun VD, Ehrlich S. [Associations between DNA methylation and schizophrenia-related intermediate phenotypes - a gene set enrichment analysis.](https://www.ncbi.nlm.nih.gov/pubmed/25598502) Prog Neuropsychopharmacol Biol Psychiatry. 59:31-9, 2015. |
| Hibar DP, Stein JL, Renteria ME, Arias-Vasquez A, Desrivières S, Jahanshad N, Toro R, Wittfeld K, Abramovic L, Andersson M, Aribisala BS, Armstrong NJ, Bernard M, Bohlken MM, Boks MP, Bralten J, Brown AA, Chakravarty MM, Chen Q, Ching CR, Cuellar-Partida G, den Braber A, Giddaluru S, Goldman AL, Grimm O, Guadalupe T, Hass J, Woldehawariat G, Holmes AJ, Hoogman M, Janowitz D, Jia T, Kim S, Klein M, Kraemer B, Lee PH, Olde Loohuis LM, Luciano M, Macare C, Mather KA, Mattheisen M, Milaneschi Y, Nho K, Papmeyer M, Ramasamy A, Risacher SL, Roiz-Santiañez R, Rose EJ, Salami A, Sämann PG, Schmaal L, Schork AJ, et al. [Common genetic variants influence human subcortical brain structures.](https://www.ncbi.nlm.nih.gov/pubmed/25607358) Nature. 9;520(7546):224-9, 2015. |
| Houck JM, Cetin MS, Mayer AR, Bustillo JR, Stephen J, Aine CJ, Canive J, Perrone-Bizzozero N, Thoma RJ, Brookes MJ, Calhoun VD. Magnetoencephalographic and functional MRI connectomics in schizophrenia via intra- and inter-network connectivity. Neuroimage In press |
| Huf W, Kalcher K, Boubela RN, Rath G, Vecsei A, Filzmoser P, Moser, E. On the generalizability of resting-state fMRI machine learning classifiers. Front Hum Neurosci. 8:502, 2014. |
| Jagannathan K, Calhoun VD, Gelernter J, Stevens MC, Liu J, Bolognani F, Windemuth A, Ruaño G, Assaf M, Pearlson GD.  [Genetic associations of brain structural networks in schizophrenia: a preliminary study.](https://www.ncbi.nlm.nih.gov/pubmed/20691427) Biol Psychiatry. 68(7):657-66, 2010. |
| Kalyanam, R, Boutte, D, Gasparovic, C, Hutchison, KE, Calhoun, VD. Group independent component analysis of MR spectra, Brain Imaging and Behavior. 3:229-242, 2013, PMC3683283. |
| Kalyanam R, Boutte D, Hutchison KE, Calhoun VD. Application of ICA to realistically simulated (1)H-MRS data. Brain Behav. 5(7):e00345, 2015. |
| King MD, Wood D, Miller B, Kelly R, Landis D, Courtney W, Wang R, Turner JA, Calhoun VD. "Automated collection of imaging and phenotypic data to centralized and distributed data repositories," Front Neuroinform. Jun5;8:60, 2014. |
| Landis D, Courtney W, Dieringer C, Kelly R, King M, Miller B, Wang RT, Wood D, Turner JA, Calhoun VD. "COINS Data Exchange: An open platform for compiling, curating, and disseminating neuroimaging data," Neuroimage*.* 124:1084-1088, 2016. |
| Lin F.-H, Tsai S-Y, Otazo R, Caprihan A, Wald LL, Belliveau JW, Posse S. Sensitivity-encoded (SENSE) Proton-Echo-Planar- Spectroscopic-Imaging (PEPSI) in Human Brain, Mag. Reson. Med. 57(2):249-257, 2007. |
| Liu J, Calhoun VD. [A review of multivariate analyses in imaging genetics.](https://www.ncbi.nlm.nih.gov/pubmed/24723883) Front Neuroinform. 8:29, 2014. |
| Liu J, Siyahhan Julnes P, Chen J, Ehrlich S, Walton E, Calhoun VD. [The association of DNA methylation and brain volume in healthy individuals and schizophrenia patients.](https://www.ncbi.nlm.nih.gov/pubmed/26381449) Schizophr Res. 169(1-3):447-52, 2015. |
| Liu J, Ulloa A, Perrone-Bizzozero N, Yeo R, Chen J, Calhoun VD. [A pilot study on collective effects of 22q13.31 deletions on gray matter concentration in schizophrenia.](http://www.ncbi.nlm.nih.gov/pubmed/23285208) PLoS One. 7(12):e52865, 2012. |
| Mayer AR, Hanlon FM, Dodd AB, Yeo RA, Haaland KY, Ling JM, Ryman SG. Proactive response inhibition abnormalities in the sensorimotor cortex of patients with schizophrenia. J Psychiatry Neurosci. 16;41(3):150097, 2016. |
| Mayer AR, Hanlon FM, Franco AR, Teshiba TM, Thoma RJ, Clark VP, Canive JM. The neural networks underlying auditory sensory gating. Neuroimage. 44(1):182-9, 2009. |
| Mayer AR, Teshiba TM, Franco AR, Ling J, Shane MS, Stephen JM, Jung RE. Modeling conflict and error in the medial frontal cortex. Hum Brain Mapp. 33(12):2843-55, 2012. |
| Mayer AR, Ruhl D, Merideth F, Ling J, Hanlon FM, Bustillo J, Cañive J. Functional imaging of the hemodynamic sensory gating response in schizophrenia. Hum Brain Mapp. 34(9):2302-12, 2013. |
| Mayer AR, Hanlon FM, Teshiba TM, Klimaj SD, Ling JM, Dodd AB, Calhoun VD, Bustillo JR, Toulouse T. An fMRI study of multimodal selective attention in schizophrenia. Br J Psychiatry. 207(5):420-8, 2015. |
| Meda SA, Jagannathan K, Gelernter J, Calhoun VD, Liu J, Stevens MC, Pearlson GD. [A pilot multivariate parallel ICA study to investigate differential linkage between neural networks and genetic profiles in schizophrenia.](https://www.ncbi.nlm.nih.gov/pubmed/19944766) Neuroimage. 53(3):1007-15, 2010. |
| Meier TB, Wildenberg JC, Liu J, Chen J, Calhoun VD, Biswal BB, Meyerand ME, Birn RM, Prabhakaran V. [Parallel ICA identifies sub-components of resting state networks that covary with behavioral indices.](https://www.ncbi.nlm.nih.gov/pubmed/23087635) Front Hum Neurosci. Oct 11, 2012;6:281. |
| Monnig MA, Caprihan A, Yeo RA, Gasparovic C, Ruhl DA, Lysne P, Bogenschutz MP, Hutchison KE, Thoma RJ. Diffusion tensor imaging of white matter networks in individuals with current and remitted alcohol use disorders and comorbid conditions. Psychol Addict Behav. 27:455-465, 2013. |
| Mounce J, Luo L, Caprihan A, Liu J, Perrone-Bizzozero NI, Calhoun VD. [Association of GRM3 polymorphism with white matter integrity in schizophrenia.](https://www.ncbi.nlm.nih.gov/pubmed/24680030) Schizophr Res. 155(1-3):8-14, 2014. |
| Mullins PG, Chen H, Xu J, Caprihan A, Gasparovic C. Comparative reliability of proton spectroscopy techniques designed to improve detection of J-coupled metabolites. Magn Reson Med. 60(4):964-9, 2008, PMID:18816817. |
| Patel VS, Kelly S, Wright C, Gupta CN, Arias-Vasquez A, Perrone-Bizzozero N, Ehrlich S, Wang L, Bustillo JR, Morris D, Corvin A, Cannon DM, McDonald C, Donohoe G, Calhoun VD, Turner JA. [MIR137HG risk variant rs1625579 genotype is related to corpus callosum volume in schizophrenia.](http://www.ncbi.nlm.nih.gov/pubmed/26123324) Neurosci Lett. 602:44-9, 2015. |
| Pearlson GD, Liu J, Calhoun VD. An introductory review of parallel independent component analysis (p-ICA) and a guide to applying p-ICA to genetic data and imaging phenotypes to identify disease-associated biological pathways and systems in common complex disorders. Front Genet 6: 276, 2015. |
| Posse S, Otazo R, Caprihan A, Bustillo J, Chen H, Henry P-G, Marjanska M, Gasparovic C, Zuo C, Magnotta V, Mueller B, Mullins P, Renshaw P, Ugurbil K, Lim KO, Alger JR, Proton Echo Planar Spectroscopic Imaging of J-Coupled Resonances in Human Brain at 3 and 4 Tesla. Magn. Reson. Med. 58(2):236–244, 2007. |
| Posse S, Otazo R, Dager SR, Alger J. Magnetic Resonance Spectroscopic Imaging: Principles and Recent Advances. Journal of Magnetic Resonance Imaging. 37(6):1301-25, 2013, PMID: 23188775. |
| Segall J, Allen EA, Jung RE, Erhardt E, Arja S, Kiehl KA, Calhoun VD. Correspondence between Structure and Function in the Human Brain at Rest," Front Neuroinform. 6:10, 2012, PMC3313067. |
| Scott A, Courtney W, Wood D, de la Garza R, Lane S, King M, Wang R, Roberts J, Turner JA, Calhoun VD. COINS: An Innovative Informatics and Neuroimaging Tool Suite Built for Large Heterogeneous Datasets. Front Neuroinform*.*5:33, 2011, 3250631. |
| Shoemaker JM, Holdsworth MT, Aine C, Calhoun VD, de La Garza R, Feldstein Ewing SW, Hayek R, Mayer AR, Kiehl KA, Petree LE, Sanjuan P, Scott A, Stephen J, Phillips JP. A practical approach to incidental finding in neuroimaging research. Neurology. 77(24):2123-7, 2011. |
| Silva RF, Castro E, Gupta CN, Cetin M, Arbabshirani M, Potluru VK, Plis SM, Calhoun VD. The tenth annual MLSP competition: Schizophrenia classification challenge. 2014 IEEE International Workshop on Machine Learning for Signal Processing, Sept. 21-24, Reims, France, 2014. |
| Stephen JM, Coffman BA, Jung RE, Bustillo JR, Aine CJ, Calhoun VD. Using joing ICA to link function and structure using MEG and DTI in schizophrenia. Neuroimage. 83:418-30, 2013. |
| Stone DB, Urrea LJ, Aine CJ, Bustillo JR, Clark VP, Stephen JM. Unisensory processing and multisensory integration in schizophrenia: a high density electrical mapping study. Neuropsychologia. 49(12):3178-87, 2011. |
| Stone DB, Coffman BA, Bustillo JR, Aine CJ, Stephen JM. Multisensory stimuli elicit altered oscillatory brain responses at gamma frequencies in patients with schizophrenia. Front Hum Neurosci. 8:788, 2014. |
| Sui J, He H, Liu J, Yu Q, Adali T, Pearlson GD, Calhoun VD. [Three-way FMRI-DTI-methylation data fusion based on mCCA+jICA and its application to schizophrenia.](https://www.ncbi.nlm.nih.gov/pubmed/23366480) Conf Proc IEEE Eng Med Biol Soc. 2012;2012:2692-5. |
| Sui J, Pearlson G, Caprihan A, Adali T, Kiehl KA, Liu J, Yamamoto J, Calhoun VD. [Discriminating schizophrenia and bipolar disorder by fusing fMRI and DTI in a multimodal CCA+ joint ICA model.](https://www.ncbi.nlm.nih.gov/pubmed/21640835) Neuroimage. 57(3):839-55, 2011. |
| Turner JA, Chen H, Mathalon DH, Allen EA, Mayer AR, Abbott CC, Calhoun VD, Bustillo J. Reliability of the amplitude of low-frequency fluctuations in resting state fMRI in chronic schizophrenia. Psychiatry Res. 201(3):253-5, 2012. |
| Ulloa A, Rodriguez P, Liu J, Calhoun V, Pattichis M. [A quasi-local method for instantaneous frequency estimation with application to structural magnetic resonance images.](https://www.ncbi.nlm.nih.gov/pubmed/25570248) Conf Proc IEEE Eng Med Biol Soc. 2014;2014:1477-80. |
| van Erp TG, Hibar DP, Rasmussen JM, Glahn DC, Pearlson GD, Andreassen OA, Agartz I, Westlye LT, Haukvik UK, Dale AM, Melle I, Hartberg CB, Gruber O, Kraemer B, Zilles D, Donohoe G, Kelly S, McDonald C, Morris DW, Cannon DM, Corvin A, Machielsen MW, Koenders L, de Haan L, Veltman DJ, Satterthwaite TD, Wolf DH, Gur RC, Gur RE, Potkin SG, Mathalon DH, Mueller BA, Preda A, Macciardi F, Ehrlich S, Walton E, Hass J, Calhoun VD, Bockholt HJ, Sponheim SR, Shoemaker JM, van Haren NE, Pol HE, Ophoff RA, Kahn RS, Roiz-Santiañez R, Crespo-Facorro B, Wang L, Alpert KI, Jönsson EG, Dimitrova R, Bois C, Whalley HC, McIntosh AM, Lawrie SM, Hashimoto R, Thompson PM, Turner JA. Subcortical brain volume abnormalities in 2028 individuals with schizophrenia and 2540 healthy controls via the ENIGMA consortium. Mol Psychiatry. 21(4):585, 2016. |
| Vergara VM, Ulloa A, Calhoun VD, Boutte D, Chen J, Liu J.[A three-way parallel ICA approach to analyze links among genetics, brain structure and brain function.](https://www.ncbi.nlm.nih.gov/pubmed/24795156) Neuroimage. 98:386-94, 2014. |
| Walton E, Geisler D, Hass J, Liu J, Turner J, Yendiki A, Smolka MN, Ho BC, Manoach DS, Gollub RL, Roessner V, Calhoun VD, Ehrlich S. [The impact of genome-wide supported schizophrenia risk variants in the neurogranin gene on brain structure and function.](https://www.ncbi.nlm.nih.gov/pubmed/24098564) PLoS One. Oct 2, 2013;8(10):e76815. |
| Wood D, King M, Landis D, Courtney W, Wang R, Kelly R, Turner JA, Calhoun VD, "Harnessing modern web application technology to create intuitive and efficient data visualization and sharing tools," Front Neuroinform*.* 8:71, 2014, 4144441. |
| Wu L, Calhoun VD, Jung RE, Caprihan A. Connectivity-based whole brain dual parcellation by group ICA reveals tract structures and decreased connectivity in schizophrenia. Hum Brain Mapp. 36(11):4681-701, 2015. PMC4619141. |
| Yeo RA, Ryman SG, van den Heuvel MP, de Reus MA, Jung RE, Pommy J, Mayer AR, Ehrlich S, Schulz SC, Morrow EM, Manoach D, Ho BC, Sponheim SR, Calhoun VD. Graph metrics of structural brain networks in individuals with schizophrenia and healthy controls: Group differences, relationships with intelligence, and genetics. J Int Neuropsychol Soc. 22(2):240-9, 2016. |
| Yeo RA, Gangestad SW, Liu J, Ehrlich S, Thoma RJ, Pommy J, Mayer AR, Schulz SC, Wassink TH, Morrow EM, Bustillo JR, Sponheim SR, Ho BC, Calhoun VD. [The impact of copy number deletions on general cognitive ability and ventricle size in patients with schizophrenia and healthy control subjects.](http://www.ncbi.nlm.nih.gov/pubmed/23237311) Biol Psychiatry. 73(6):540-5, 2013. |
| Yeo RA, Gangestad SW, Walton E, Ehrlich S, Pommy J, Turner JA, Liu J, Mayer AR, Schulz SC, Ho BC, Bustillo JR, Wassink TH, Sponheim SR, Morrow EM, Calhoun VD. [Genetic influences on cognitive endophenotypes in schizophrenia.](http://www.ncbi.nlm.nih.gov/pubmed/24768440) Schizophr Res. 156(1):71-5, 2014. |
| Yeo RA, Martinez D, Pommy J, Ehrlich S, Schulz SC, Ho BC, Bustillo JR, Calhoun VD. [The impact of parent socio-economic status on executive functioning and cortical morphology in individuals with schizophrenia and healthy controls.](http://www.ncbi.nlm.nih.gov/pubmed/23866983) Psychol Med. 44(6):1257-65,2014. |
| Yu Q, Wu L, Bridwell DA, Erhardt EB, Du Y, He H, Chen J, Liu P, Sui J, Pearlson G, Calhoun VD. [Building an EEG-fMRI Multi-Modal Brain Graph: A Concurrent EEG-fMRI Study.](https://www.ncbi.nlm.nih.gov/pubmed/27733821) Front Hum Neurosci. Sep 28;10:476, 2016. |
| Yu Q, Sui J, Liu J, Plis SM, Kiehl KA, Pearlson G, Calhoun VD. Disrupted correlation between low frequency power and connectivity strength of resting state brain networks in schizophrenia. Schizophr Res. 143(1):165-71, 2013. |
| **Table 2.** Publications resulting from use of Phase I COBRE data and/or core resources. |
